# Advanced modes of mechanical ventilation and optimal targeting schemes

**DOI:** 10.1186/s40635-018-0195-0

**Published:** 2018-08-22

**Authors:** Matthias van der Staay, Robert L. Chatburn

**Affiliations:** 1 IMT Information Management Technology, Gewerbestrasse 8, 9470 Buchs, Switzerland; 2 Respiratory Institute, Cleveland Clinic, Cleveland, OH USA

**Keywords:** Mechanical ventilation, Mathematical modeling, Lung protective ventilation, Optimal targeting schemes, Simulation

## Abstract

Recent research results provide new incentives to recognize and prevent ventilator-induced lung injury (VILI) and create targeting schemes for new modes of mechanical ventilation. For example, minimization of breathing power, inspiratory power, and inspiratory pressure are the underlying goals of optimum targeting schemes used in the modes called adaptive support ventilation (ASV), adaptive ventilation mode 2 (AVM2), and MID-frequency ventilation (MFV). We describe the mathematical models underlying these targeting schemes and present theoretical analyses for minimizing tidal volume, tidal pressure (also known as driving pressure), or tidal power as functions of ventilatory frequency. To go beyond theoretical equations, these targeting schemes were compared in terms of expected tidal volumes using different patient models. Results indicate that at the same ventilation efficiency (same PaCO_2_ level), we expect tidal volume dosage in the range of 7.4 mL/kg (for ASV), 6.2 mL/kg (for AVM2), and 6.7 mL/kg (for MFV) for adult ARDS simulation. For a neonatal RDS model, we expect 5.5 mL/kg (for ASV), 4.6 mL/kg (for AVM2), and 4.5 (for MFV).

**Electronic supplementary material:**

The online version of this article (10.1186/s40635-018-0195-0) contains supplementary material, which is available to authorized users.

## Background

Adaptive ventilation modes are designed to automate some of the basic actions of clinicians as they attempt to identify the best settings, although the definition of “best” continues to be a matter of debate. The algorithms usually adapt to the changing characteristics of the patient, such as mechanics (resistance, compliance, and inspiratory effort) or ventilatory pattern (frequency and tidal volume), and choose an appropriate response. One strategy to incorporate clinical knowledge into machine design is to use what is called an optimum targeting scheme [1], a term adapted from engineering control theory. An optimum targeting scheme is based on a mathematical model that attempts to minimize or maximize some desired outcome. In optimization theory, that model is also called a cost function. This function tells the machine how much a ventilation pattern “costs” in terms of predefined criteria. These criteria are based on actual patient characteristics (e.g., the cost function could simply describe tidal volume dosage). Hence, the goal of an optimum targeting scheme is to find the ventilation pattern with the lowest cost. If this optimum pattern is found, it can be used to set values (targets) for the underlying controllers. For a detailed description of how optimal targeting schemes work, see the Additional file 1.

In the first section of this paper, familiar ventilation parameters (tidal volume, tidal pressure, and tidal power) are used to derive cost functions. Also, the cost functions, which underlie the ventilation modes called adaptive support ventilation (ASV), adaptive ventilation mode 2 (AVM2), and mid frequency ventilation (MFV), are derived. Next, we perform mathematical analyses to compare the characteristics of these optimum target schemes. These analyses tend to be complex and hard to interpret intuitively. For this reason, a summary of clinical evidence is presented along with mathematical simulations we performed to compare and visualize the results of the cost function minimization.

**Fig. 1:**
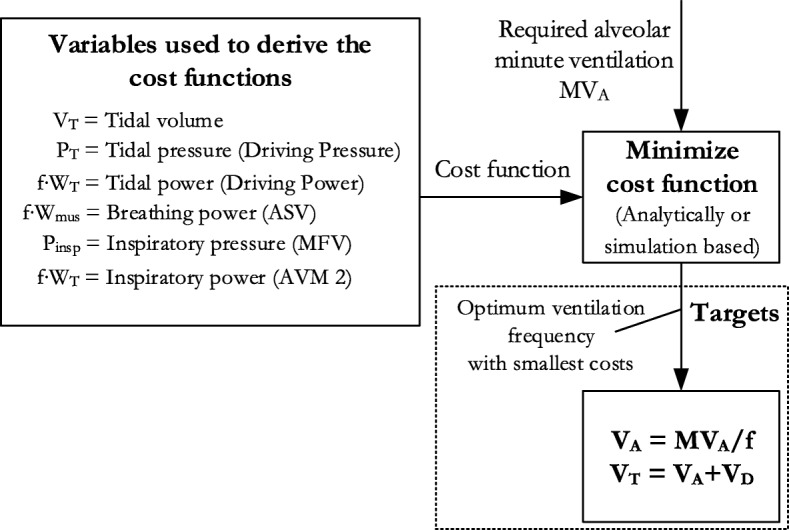
Relation between cost functions and targets. Every cost function (tidal volume, tidal pressure, tidal power, breathing power, inspiratory power, and inspiratory pressure) was minimized analytically or using mathematical simulation. The result is the “optimal” ventilation frequency, which reaches the set alveolar minute ventilation and minimizes the cost function. After that, the frequency was used to calculate the target tidal volume

Figure 1 shows the variables used to derive the cost functions and how cost functions are used to optimize the ventilatory pattern.

## Methods

### What should we optimize?

#### Tidal volume

The pivotal study by the Acute Respiratory Distress Syndrome Network in 2000 established the notion that in patients with acute lung injury and acute respiratory distress syndrome, mechanical ventilation with a lower tidal volume dosage (6.2 vs 11.8 mL/kg ideal body weight) decreases mortality and increases the number of ventilator-free days [2]. There are also data to support the use of low *V*_T_ in patients without pre-existing lung injury [3–6]. A recent study even suggests that lung protective ventilation might be considered a prophylactic therapy, rather than just a supportive therapy [7].

If we assume a value for the required alveolar minute volume (MV_A_) and simply desire to control the tidal volume (*V*_T_) dosage for a passive patient, we can derive the cost function as follows:

1 $$ {V}_{\mathrm{T}}=\frac{{\mathrm{MV}}_{\mathrm{A}}}{f}+{V}_{\mathrm{D}} $$

where *V*_D_ represents the dead space volume and *f* the ventilatory frequency, and hence, MV_A_/*f* represents the alveolar volume. Thus, the “cost” in terms of tidal volume dosage (and presumably the risk of VILI) goes down as frequency goes up for a given required minute alveolar ventilation. However, we see that there is no definite minimum value because tidal volume converges to the dead space volume as frequency increases to infinity. In practice, the limit would be dependent on the volume delivery performance characteristics of the ventilator, because no ventilator is a perfect flow controller. Also, in the USA, conventional ventilator frequency is limited to a maximum of 150 breaths/minute.

#### Tidal pressure

Simply controlling the tidal volume dosage, independent of any consideration of lung mechanics, may have limited utility. Recent work has suggested that *V*_T_ normalized to lung mechanics (e.g., *V*_T_/C) is a better predictor of mortality than tidal volume dosage [8–10]. We prefer to call *V*_T_/*C* (or equivalently, *P*_plat_ – totalPEEP) tidal pressure, *P*_T_, instead of driving pressure because *P*_T_ differs from *V*_T_ by only a scaling factor and driving pressure is sometimes used in reference to any pressure driving flow, not just static end-inspiratory pressure at the airway opening. In a cohort of brain-injured patients, *P*_T_ was associated with the development of ARDS [11]. In a series of ARDS patients receiving ECMO for refractory hypoxemia, *P*_T_ during ECMO was the only ventilator setting that showed an independent association with in-hospital mortality [12]. In patients having surgery, intra-operative high *P*_T_ and changes in the level of PEEP that resulted in an increase of *P*_T_ were associated with more postoperative pulmonary complications [13]. On the other hand, if *V*_T_ is strictly maintained at 6 mL/kg predicted body weight and *P*_plat_ below 28–30 cm H_2_O, then *P*_T_ shares the same information as *P*_plat_ about the association with day 90-mortality [14].

However, if we define an optimal targeting scheme as minimization of *P*_T_, we get the same result as minimizing to tidal volume because the tidal pressure is linked to driving pressure by compliance, *C*, which can be considered simply a scaling factor. If compliance only affects the scaling of the cost function, then it has no influence to the location of the minimum.

2 $$ {P}_{\mathrm{T}}=\frac{1}{C}\cdot \left(\frac{{\mathrm{MV}}_{\mathrm{A}}}{f}+{V}_{\mathrm{D}}\right) $$

#### Tidal power

Gattinoni et al. have suggested an association between power transfer (from ventilator to lungs) and VILI [15]. However, as Marini and Jaber have observed [16] “…it is difficult to link power dissipated in proximal airway resistance directly to noxious events at the alveolar level.” Furthermore, they discount the effect of PEEP on the power equation because “…the ventilator’s work against PEEP is temporarily stored as potential energy within the elastic tissues of the respiratory system; it later converts to kinetic energy as the gas escapes to atmosphere across the exhalation valve.” Hence, the power used to deliver the tidal volume against PEEP is not stored in the body and would not be expected to contribute to lung injury. Thus, they have suggested that a potentially better indicator of injury risk for clinical purposes might be “driving power” defined as:

3 $$ \mathrm{Driving}\kern0.50em \mathrm{power}=\frac{f\cdot {V}_{\mathrm{T}}\cdot {P}_{\mathrm{T}}}{10\cdot C} $$

where *C* (compliance) is a scaling factor used to account for “…the reduced capacity of the ‘baby lung’.” However, we can define tidal power as

4 $$ \mathrm{Tidal}\kern0.50em \mathrm{power}=\frac{f\cdot {V_{\mathrm{T}}}^2}{2\cdot C}=\frac{f\cdot {V}_{\mathrm{T}}\cdot {P}_{\mathrm{T}}}{2} $$

which is equal to total power without the resistive portion and the energy which escapes to atmosphere during expiration. Marini and Jaber suggested driving power as a metric that could be associated with the risk of VILI and recommended that power be normalized “…at least for aerated lung capacity.” If tidal power is used as the cost function, we replace *V*_T_ in Eq. (4) with Eq. 1 and then solve the following optimization problem:

5 $$ \underset{f\in \left[0,\infty \right]}{\arg \min}\frac{f}{2\cdot C}\cdot {\left(\frac{{\mathrm{MV}}_{\mathrm{A}}}{f}+{V}_{\mathrm{D}}\right)}^2 $$

The solution is obtained analytically by differentiating tidal power with respect to *f* and setting the result to zero. Solving Eq. 5 for optimal frequency leads to the remarkable result of

6 $$ {f}_{\mathrm{TP}}=\frac{{\mathrm{MV}}_{\mathrm{A}}}{V_{\mathrm{D}}}=\frac{\mathrm{MV}}{2\cdot {V}_{\mathrm{D}}} $$

where *f*_TP_ = frequency of minimum tidal power and MV = minute volume measured at the proximal airway. If we express MV as the product of tidal volume and frequency, the optimal tidal volume (i.e., optimal in terms of minimal tidal power) can be expressed simply as function of dead space:

7 $$ {V}_T=2\cdot {V}_D $$

Furthermore, if we assume *V*_D_ = 2.2 mL/kg (IBW) as an estimation for normal dead space volume, the tidal volume would be given by:

8 $$ {V}_{\mathrm{T}}=4.4\ \mathrm{mL}/\mathrm{kg} $$

for minimal tidal power to ventilate normal lungs. As mentioned, driving power is connected by a scaling factor to tidal power. Therefore, the condition of minimal driving power is fulfilled at the same optimal frequency and therefore yields the same optimal tidal volume.

Cressoni et al. defined transpulmonary mechanical work as the area between the inspiratory limb of the transpulmonary pressure vs volume curve during inspiration with constant flow [17]. They showed that if transpulmonary mechanical power (work per breath times respiratory frequency) exceeded the limit of 12 J/min, five out of five piglets developed whole-lung edema and four out of four did not when they were ventilated below that threshold.

#### Breathing power (Adaptive Support Ventilation)

In 1950, Otis et al. investigated unassisted breathing frequency with respect to lung mechanics and alveolar minute ventilation [18]. They made the assumption that the brain seeks an optimum frequency by minimizing breathing effort. To derive the cost function of breathing effort, they assumed a one compartment lung model with linear compliance and non-linear resistance:

9 $$ {P}_{\mathrm{mus}}(t)=\frac{1}{C}\cdot V(t)+R\cdot \dot{V}(t)+{R}^{\prime}\cdot \dot{V}{(t)}^2 $$

where *R* is the linear (viscous) and *R*′ the non-linear (turbulent) portion of airway resistance and *P*_mus_ the pressure generated by inspiratory muscles. The flow $\dot{V}$ was assumed to follow a sine curve with an *I*:*E* ratio of 1:1:

10 $$ \dot{V}(t)=\hat{\dot{V}}\cdot \sin \left(2\cdot \uppi \cdot f\cdot t\right) $$

where $\widehat{\dot{V}}$ represents the peak flow. On the basis of that model, breathing effort was defined as work rate or power. With the assumptions of Eqs. 9 and 10, the mean rate of muscular work was derived as [18]:

11 $$ \underset{\mathrm{Totalpower}}{\underbrace{{\dot{W}}_{\mathrm{mus}}}}=\underset{\mathrm{Tidalpower}}{\underbrace{\frac{f}{2\cdot \mathrm{C}}\cdot {\left(\frac{{\mathrm{MV}}_{\mathrm{A}}}{f}+{V}_{\mathrm{D}}\right)}^2}}+\underset{\mathrm{Resistivepower}\left(\mathrm{viscous}\right)}{\underbrace{\frac{1}{4}\cdot R\cdot {\uppi}^2\cdot {\left({\mathrm{MV}}_{\mathrm{A}}+f\cdot {V}_{\mathrm{D}}\right)}^2}}+\underset{\mathrm{Resistivepower}\left(\mathrm{turbulent}\right)}{\underbrace{\frac{2}{3}\cdot {R}^{\prime}\cdot {\uppi}^2\cdot {\left({\mathrm{MV}}_{\mathrm{A}}+f\cdot {V}_{\mathrm{D}}\right)}^3}} $$

To find the optimal frequency at minimal breathing power, the following optimization problem must be solved.

12 $$ \underset{f\in \left[0,\infty \right]}{\arg \min }{\dot{W}}_{\mathrm{mus}} $$

Otis solved Eq. 12 by differentiating Eq. 11 with respect to *f* and setting the result equal to zero. Instead of solving for *f*, he solved the equation for MV_A_ to get a solution for the conditions of minimal breathing power. Later, Mead [19] simplified Eq. 9 by neglecting the term with turbulent flow resistance (${R}^{\prime}\cdot \dot{V}{(t)}^2$). Then, he solved the optimization problem of Eq. (12), resulting in the well-known equation for determining the optimal frequency at minimal breathing power (*f*_BP_):

13 $$ {f}_{\mathrm{BP}}=\frac{-1+\sqrt{1+\frac{4\cdot {\uppi}^2\cdot \mathrm{RC}\cdot {\mathrm{MV}}_{\mathrm{A}}}{V_{\mathrm{D}}}}}{2\cdot {\uppi}^2\cdot \mathrm{RC}} $$

As an alternative to Eq. (13), Mead also showed that an optimal frequency exists at which the average force per breath required from the respiratory muscles is minimal (*f*_BF_):

14 $$ {f}_{\mathrm{BF}}={\left(\frac{{\mathrm{MV}}_{\mathrm{A}}}{V_D}\right)}^{1/3}\cdot {\left(2\uppi \mathrm{RC}\right)}^{-2/3} $$

Otis et al. and Mead derived their equations to better understand the energetics of breathing and the associated effects on “the imaginary path from health to disease.” They were not concerned with inventing new modes of mechanical ventilation.

In 1991, Fleur T. Tehrani patented a targeting scheme based on Eq. 13. The system was designed to “…reduce the load on the respiratory muscles, mimic natural breathing, stimulate spontaneous breathing, and reduce weaning time” [20]. Interestingly, the initial implementation of this targeting scheme was not to minimize power delivery from ventilator to patient [21], but rather to select initial settings and “…choose a breathing pattern that encourages the patients to breathe on their own as early as possible” [22]. Note that the development of this targeting scheme was almost a decade before intensive research on the role of tidal volume dosage on mortality. At that time, the concern was to avoid an excessively large tidal volume, not to minimize it. Nevertheless, over the years, ASV has proven to be effective and results in relatively protective tidal volume delivery in the range of 8.1 ± 1.4 mL/kg ideal body weight [23].

#### Inspiratory power (Adaptive Ventilation Mode 2)

Ventilation modes using adaptive targeting based on Eq. 13 do not necessarily deliver lung protective ventilation [24, 25]. To reduce tidal volume (and subsequently tidal pressure) [10], we can derive the concept of mean inspiratory power [26]. Inspiratory power is defined as the sum of the resistive and tidal power which is transmitted from the ventilator to the patient assuming intrinsic PEEP equal zero:

15 $$ \mathrm{Inspiratory}\ \mathrm{power}\ \left({\dot{W}}_{\mathrm{insp}}\right)=\mathrm{tidal}\ \mathrm{power}\ \left({\dot{W}}_{\mathrm{T}}\right)+\mathrm{resistive}\ \mathrm{power}\ \left({\dot{W}}_{\mathrm{R}}\right) $$

**Fig. 2:**
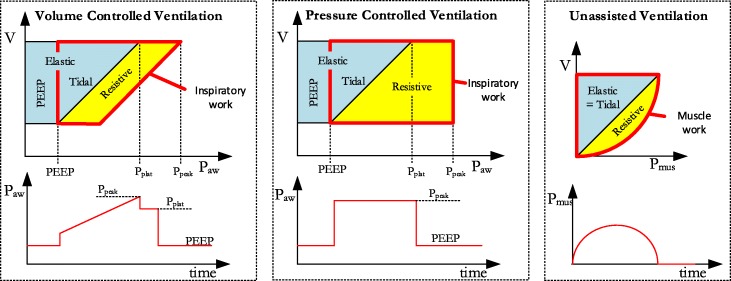
Definition of the different power components of inspiration and expiration. Breathing power was introduced by Otis, resistive and elastic power was defined by Gattinoni, and Marini differentiated elastic power into its components PEEP power and tidal power. The authors now introduce the concept of inspiratory power which is composed of tidal and resistive power. Note that the figure shows work instead of power and power is the result of the product between work and ventilation frequency

There are differences among inspiratory power, total power [15], elastic power, breathing power, and tidal power. Elastic power includes tidal power and PEEP power, inspiratory power includes tidal power and resistive power, and total power includes elastic power and resistive power. Figure 2 and Table 1 explain these concepts (which were created by Otis, Gattinoni, Marini, and us). Note that power is defined as the work per unit time, which is calculated as the product of work and ventilatory frequency. Inspiratory work per breath is defined as the integral of inspiratory pressure with respect to inspiratory volume, or graphically, the area between the pressure curve and the volume axis as shown in Fig. 2.

**Table 1:** Definition of the different types of power relating to Fig. 2

Total work:	Work generated by the ventilator to deliver pressure and flow to the respiratory system.
Resistive work:	Work dissipated (as heat) in generating flow through the airway resistance
Elastic work:	Work needed to expand the lungs and chest wall.
PEEP work:	Work against PEEP during inspiration which is temporarily stored as energy within the elastic tissues. It later converts to kinetic energy as the gas escapes to atmosphere across the exhalation valve.
Tidal work:	Elastic work minus PEEP work.
Inspiratory work:	The sum of tidal work and resistive work.
Muscle work:	Work generated by the ventilatory muscles.

There is an important difference between *muscle* power ${\dot{W}}_{\mathrm{mus}}$ and *inspiratory* power. Otis derived the mean power which is needed to breathe *without* the support of a ventilator with a *sinusoidal muscle pressure waveform*. On the contrary, the concept of inspiratory power relies on the principle of how much power is delivered to the patient by a *ventilator using a square pressure waveform* (assuming total PEEP equals zero).

Inspiratory power is not intended to be another predictor for VILI. Instead, it serves as the basis for defining an alternative cost function which may be used to describe an optimal ventilation pattern. Inspiratory power includes not only tidal power (which might be a better indicator for VILI) but also includes resistive power. This leads to a more “natural” ventilation similar to Otis’ breathing power. However, as we will see, minimizing inspiratory power converges for specific patient characteristics to the same result as minimizing tidal power which might be relevant for VILI prevention.

The derivation of inspiratory power for pressure controlled ventilation of a one-compartment linear lung model is provided in the Additional file 1. Inspiratory power can be calculated as:

16 $$ {\dot{W}}_{\mathrm{insp}}=\frac{1}{2\cdot C}\cdot f\cdot {\left(\frac{{\mathrm{MV}}_{\mathrm{A}}}{f}+{V}_{\mathrm{D}}\right)}^2\cdot \left(1+\coth \left(\frac{T_{\mathrm{I}}}{2\cdot R\cdot C}\right)\right) $$

where coth() is the cotangens hyperbolicus function and *T*_I_ is the set inspiratory time on the ventilator. To find the optimal frequency for minimal inspiratory power *f*_IP_, the following optimization problem must be solved:

17 $$ \underset{f\in \left[0,\infty \right]}{\arg \min }{\dot{W}}_{\mathrm{insp}} $$

Assuming that *I*:*E* = 1:1, the following numerical solution can be derived:

18 $$ {f}_{\mathrm{IP}}=\frac{\mathrm{MV}}{2\cdot {V}_{\mathrm{D}}}\left(1-\frac{1}{2\cdot {f}_{\mathrm{IP}}\cdot R\cdot C\cdot \left({e}^{\frac{1}{2\cdot {f}_{\mathrm{IP}}\cdot R\cdot C}}-1\right)}\right) $$

Note that Eq. 18 is a so-called “fixed point iteration.” That means we cannot directly calculate the optimal frequency *f*_IP_. The optimum frequency is found using an iterative numerical process, starting with a seed value. It can be shown that Eq. 18 converges to the solution for minimal tidal power of Eq. 6 for small respiratory system time constants (see Additional file 1). Therefore, *the optimal frequency for minimal inspiratory power is always equal to or less than the frequency for minimal tidal power*.

19 $$ {f}_{\mathrm{IP}}\le {f}_{\mathrm{TP}} $$

#### Inspiratory pressure (Mid Frequency Ventilation)

Marini et al. derived an equation which allows prediction of tidal volume in terms of ventilator settings and lung mechanics [27]. In 2013, Chatburn and Mireles-Cabodevila extended this equation to predict alveolar minute volume as a function of frequency and invented a new optimal targeting scheme called mid-frequency ventilation (MFV) [28]. MFV is designed to maximize alveolar minute ventilation for a given inspiratory pressure target [28] or minimize inspiratory pressure target for a target minute alveolar ventilation [29] (inspiratory pressure target, *P*_insp_, is the preset pressure change above PEEP set on the ventilator, i.e., the amplitude of the square pressure waveform).

According to Marini, tidal volume can be expressed as:

20 $$ {V}_T={P}_{\mathrm{insp}}\cdot C\cdot \frac{\left(1-{e}^{-\frac{D}{f\cdot {R}_{\mathrm{I}}\cdot C}}\right)\cdot \left(1-{e}^{-\frac{1-D}{f\cdot {R}_{\mathrm{E}}\cdot C}}\right)}{\left(1-{e}^{-\frac{D}{f\cdot {R}_{\mathrm{I}}\cdot C}}\cdot {e}^{-\frac{1-D}{f\cdot {R}_{\mathrm{E}}\cdot C}}\right)} $$

where *R*_I_ is the inspiratory resistance, *R*_E_ the expiratory resistance, and *D* the fraction of Ti to the period *T*. The ventilation frequency *f* is denoted in hertz. With a constant dead space volume *V*_*D*_, the alveolar minute volume is given by:

21 $$ {\displaystyle \begin{array}{cc}{\mathrm{MV}}_{\mathrm{A}}& =f\cdot \left({V}_{\mathrm{T}}-{V}_{\mathrm{D}}\right)\ \\ {}& =f\cdot \left[{P}_{\mathrm{insp}}\cdot C\cdot \frac{\left(1-{e}^{-\frac{D}{f\cdot {R}_{\mathrm{I}}\cdot C}}\right)\cdot \left(1-{e}^{-\frac{1-D}{f\cdot {R}_{\mathrm{E}}\cdot C}}\right)}{\left(1-{e}^{-\frac{D}{f\cdot {R}_{\mathrm{I}}\cdot C}}\cdot {e}^{-\frac{1-D}{f\cdot {R}_{\mathrm{E}}\cdot C}}\right)}-{V}_{\mathrm{D}}\right]\end{array}} $$

To get the optimal ventilation frequency, the following equation must be solved to maximize alveolar minute volume:

22 $$ \underset{f\in \left[0,\infty \right]}{\arg \max }{\mathrm{MV}}_{\mathrm{A}} $$

This can be solved experimentally by trying out different frequencies [28]. Equation 21 can be also used in a different way to minimize inspiratory pressure under the condition of constant minute volume. Hence, it can be used in the same way as the equations of ASV or AVM2. Consequently, we are able to find the minimal required set inspiratory target pressure for a desired alveolar minute volume. The frequency of minimal inspiration pressure (*P*_insp_) can be found by solving the following optimization problem:

23 $$ \underset{f\in \left[0,\infty \right]}{\arg \min }{P}_{\mathrm{insp}} $$

where *P*_insp_ can be expressed by rearranging Eq. (21) as:

24 $$ {P}_{\mathrm{insp}}\kern0.5em =\underset{\mathrm{Tidalpressure}}{\underbrace{\frac{{\mathrm{MV}}_{\mathrm{A}}+f\cdot {V}_{\mathrm{D}}}{f\cdot C}}}\cdot \frac{\left(1-{e}^{-\frac{D}{f\cdot {R}_{\mathrm{I}}\cdot C}}\cdot {e}^{-\frac{1-D}{f\cdot {R}_{\mathrm{E}}\cdot C}}\right)}{\left(1-{e}^{-\frac{D}{f\cdot {R}_{\mathrm{I}}\cdot C}}\right)\cdot \left(1-{e}^{-\frac{1-D}{f\cdot {R}_{\mathrm{E}}\cdot C}}\right)} $$

Equation 23 can be also solved experimentally by substituting different values for ventilation frequency in Eq. 24. Logically, the same frequency which maximizes alveolar minute volume will also minimize inspiratory pressure.

## Evidence for optimum targeting schemes

Note that the Additional file 1 contains extensive tables summarizing the published evidence in simulations, animal studies, and human studies.

Much has been written about ASV (note that IntelliVent ASV is an advanced variety of ASV, with the same taxonomic mode classification but with the addition of automatic control of minute ventilation target, PEEP, and FiO_2_). In preparing this manuscript, a Google search using the term “Adaptive Support Ventilation” revealed 72 references between 2000 and 2017. ASV evolved as a form of the mode called Mandatory Minute Volume described by Hewlett et al. in 1977 [30].

Mireles-Cabodevila and Chatburn [28] introduced MFV in 2008. They used an interactive mathematical model of ventilator output during pressure control ventilation (implemented as MFV) to predict the frequency at which alveolar ventilation is maximized with the lowest tidal volume for a given inspiratory pressure target. The results of the mathematical simulation were verified with a mechanical breathing simulator connected to five different ventilators. MFV allowed the use of lower inspiratory pressures and tidal volumes than conventional management of pressure control ventilation while maintaining adequate simulated gas exchange. Recently, a randomized controlled trial compared MFV to volume targeted ventilation (i.e., pressure control ventilation with adaptive targeting). Again, inspiratory pressures and tidal volumes were lower during mid-frequency ventilation [29].

AVM2 was announced in 2017 [26] when van der Staay and Remus compared AVM2 with AVM and ASV using a lung simulator modeling a patient with restrictive lung disease. They demonstrated that minimizing inspiratory power (AVM2) results in higher frequencies, lower inspiratory pressure targets, and lower *V*_*T*_ compared with minimizing breathing power (AVM and ASV). The tidal volume dosage dropped from 7 to 5.3 mL/kg. However, there are currently no animal or human studies available with AVM2.

## Results

### Theoretical comparison of ASV, MFV, and AVM2

The optimal targeting schemes described above have one thing in common: for a given required alveolar minute ventilation and set of lung mechanics (for passive ventilation), they all suggest a ventilatory frequency that is optimal in some way (along with the associated optimal tidal volume). For a given alveolar minute ventilation, there are only two parameters which are free to vary (alveolar volume and frequency), and there are virtually infinite combinations. These targeting schemes offer three different ways to make the optimal selections. ASV is based on a model of *unassisted* breathing (sinusoidal pressure waveform driving function), under the assumption that the optimal tidal volume and frequency are those that would be picked by the patient’s brain to minimize power output of the muscles. AVM2 is based on a model of *assisted* breathing (square pressure waveform driving function) and selects tidal volume and frequency such that inspiratory power will be minimized to possibly avoid ventilator-induced lung injury. Likewise, MFV was invented with the intention of serving lung protective ventilation by choosing the frequency that minimizes inspiratory pressure.

Currently, there is intensive research in the field of ventilation-induced lung injury, but definitive answers are still pending [31]. A further complication is the fact that the goals of mechanical ventilation (safety, comfort, and liberation) are often mutually exclusive. Hence, we did not seek to rate the performance of the optimum targeting schemes in terms of clinical outcomes because this is not possible without further experimental evidence. Instead, we determined how these targeting schemes behaved during selected simulation scenarios. This may promote understanding on a more intuitive level, instead of analyzing abstract mathematical equations.

#### Simulation parameters

We performed a comparison of the three targeting schemes by assuming four different mathematical simulation scenarios. The values of resistance and compliance were based on the work of Arnal et al. [32] for adult patients and of McCann et al. [33] for the neonatal scenario. With regard to dead space (*V*_*D*_), there are several options. The physiological dead space, based on the Bohr equation (*V*_D-B_), is consistent with the equation *V*_T_ = *V*_A_ + *V*_D_. [34]. ASV and AVM2 targeting schemes assume the dead space to be anatomical (*V*_D-A_), which is estimated to be 2.2 mL/kg [35] among all patient types regardless of the disease condition. In the ICU, *V*_*D*_/*V*_*T*_ calculated with the blood gas measurement of PaCO_2_ is a useful indicator for the efficiency of ventilation. In this case, the dead space volume is calculated with the Enghoff modification of the Bohr equation (*V*_D-E_). Note that this volume does not necessarily exist physically and usually overestimates the physiological dead space volume (thus, it underestimates the required MV_A_) [36]. To be clear, the presence of shunt and low $\dot{V}/\dot{Q}$ are not dead space volumes but their effects manifest in the form of “virtual” dead space (the difference between *V*_D-E_ and *V*_D-B_). Hence, if we want to simulate clinical experience using actual modes, we use *V*_D-A_ for the frequency calculation of ASV and AVM2, respectively, and *V*_D-E_ for MFV. However, we want to compare these targeting schemes at the same level of simulated PaCO_2_. For this reason, we define the “Enghoff alveolar minute volume” as

25 $$ {\mathrm{MV}}_{\mathrm{A}-\mathrm{E}}=f\cdot \left({V}_{\mathrm{T}}-{V}_{\mathrm{D}-\mathrm{E}}\right) $$

which may underestimate the alveolar minute volume MV_A_ but is best correlated to PaCO_2_. This reflects practical ventilation performance and efficiency as realistically as possible.

For the adult ARDS simulation, we assumed *V*_D-E_ = 4.4 mL/kg [37], for normal adults *V*_D-E_ = 2.2 mL/kg, and for adults with COPD *V*_D-E_/*V*_T_ = 0.49 [38] at *V*_T_/kg = 8.9 mL/kg [32] which results also in *V*_D-E_ = 4.4 mL/kg. For the neonate simulation, we assumed that a normal *V*_D-E_ = 2.5 mL/kg [33], for RDS *V*_D-E_ = 3.8 mL/kg [33], and for chronic lung disease (CLD) *V*_D-E_ = 3.8 mL/kg [33].

The parameters of the lung models are listed in Table 2. Furthermore, we assumed passive inspiration and a linear, single compartment lung model with equal inspiratory and expiratory airway resistance. The ventilator inspiration to expiration ratio was assumed to be 1:1 because Otis’ model of breathing power is based on this ratio. Accordingly, it was ensured that the results are comparable among the different targeting schemes. With these assumptions, we calculated the optimum frequency with Eq. (13) for ASV, Eq. (18) for AVM2, and Eq. (23) for MFV and then derived tidal volume (*V*_T_ = MV_A_/*f* + *V*_D_), tidal pressure (*V*_T_/*C*), tidal power (*f·V*_T_·*P*_T_/2), and Enghoff alveolar minute volume MV_A-E_ by Eq. (25).

**Table 2:** Patient characteristics for different scenarios

Scenario	Ideal weight (kg)	*R* (cmH_2_O /L/s)	*C* (ml/cmH_2_O)	*V*_D-A_ (ml)	*V*_D-E_ (ml)
Adult:	70	10	55	154	154
Adult COPD:	70	20	60	154	308
Adult severe ARDS:	70	10	35	154	308
Neonatal:	1	37	1.5	2.2	2.5
Neonatal RDS:	1	120	0.4	2.2	3.8
Neonatal CLD:	1	90	0.5	2.2	3.8

The anatomic dead space *V*_D-A_ was used for the frequency calculation of ASV and AVM2. *V*_D-E_ represents the dead space determined by PaCO_2_ and the Enghoff modification of the Bohr equation. For the simulation, this was used both for the frequency calculation of MFV and for the calculation of the Enghoff alveolar minute volume MV_A-E_

Calculation of the optimum frequency for the MFV target scheme was done experimentally using different simulated ventilator frequency setting values. We chose an interval of 0.1 breaths per minute to avoid excessive error. The same precision was applied for the iterative calculation of the ASV and AVM2 frequency. The simulation was done with the software package Matlab from Mathworks and comprises the following steps:

- Set target alveolar minute volume (MV_A_)
- Calculate and find the optimal frequency for ASV, AVM2, and MFV by the equations of the first section and the parameters defined above.
- Calculate the resultant ventilation parameters of tidal volume, tidal pressure, tidal power, inspiratory power, and Enghoff alveolar minute volume for comparison.
- Repeat steps (a) to (c) for different MV_A_ targets

#### Adults

**Fig. 3:**
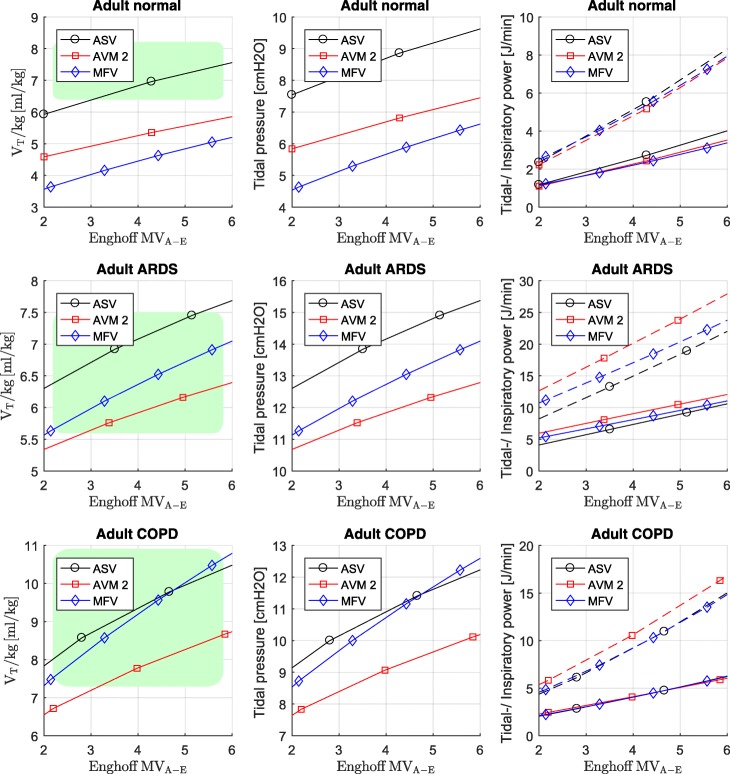
Simulation results of adult normal, severe ARDS, and COPD. Data points are optimum values for tidal volume, tidal pressure, tidal power (solid lines), and inspiratory power (dashed lines) for various levels of Enghoff alveolar minute ventilation (MV_A-E_). The green area highlights the area which is normally used according to the work of Arnal. For COPD, ASV and MFV have similar results. For ARDS simulation scenario, the concept of AVM2 yields the lowest tidal volumes and the concept of ASV uses highest tidal volumes

For adult 70 kg simulation, we compare normal, ARDS, and COPD lung characteristics according to Table 2. Results are shown in Fig. 3. The green area highlights the range which is normally used for these patients according to Arnal et al. [32].

#### Neonatal

**Fig. 4:**
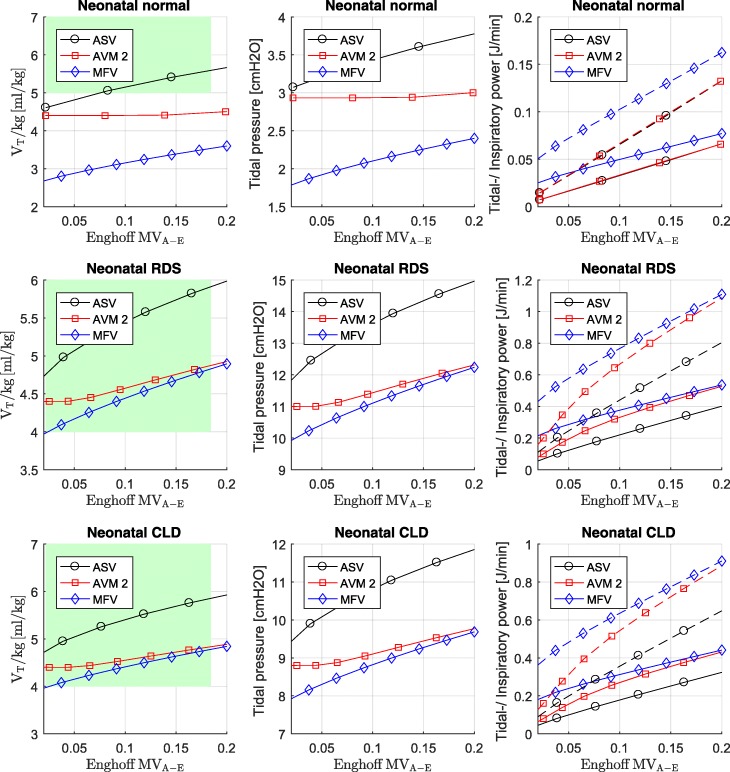
Simulation results of neonatal normal, RDS, and CLD. Data points are optimum values for tidal volume, tidal pressure, tidal power (solid lines), and inspiratory power (dashed lines) for various levels of Enghoff alveolar minute ventilation (MV_A-E_). The green area highlights the area which is normally used according to the work of McCann. For AVM2, it can be seen that the V_T_/kg ratio does not fall below 2·V_D-A_, which is the equivalent for minimal tidal power. For RDS, CLD, and normal simulation scenario, the concept of MFV yields the lowest tidal volumes and the concept of ASV yields the highest tidal volumes

For neonatal simulation, we compare normal, RDS, and CLD lung characteristics according to Table 2. Results are shown in Fig. 4. The green areas highlight the range which is normally used for these patients according to McCann et al. [33].

## Discussion

### The effect of dead space

An attentive observer of Fig. 3 and Fig. 4 will notice that AVM2, which optimizes by minimizing inspiratory power, does not always have the lowest inspiratory power. On the other hand, MFV, which optimizes by minimizing inspiratory pressure, does not always have the smallest tidal pressure. These findings can be explained by the mismatch of dead space *V*_D_. For ASV and AVM2, the calculation of the optimal frequencies is based on *V*_D-A_ and not on *V*_D-E_. Therefore, if *V*_D-A_ is significantly lower than *V*_D-E_, the Enghoff alveolar minute volume is underestimated. To get the same Enghoff alveolar minute volume, we increased the alveolar minute volume until the desired Enghoff alveolar minute volume was reached (resulting in the same simulated PaCO_2_ level). Hence, the calculation for the optimal frequencies for ASV, AVM2, and MFV cannot be done at the same alveolar minute volume to reach the same Enghoff alveolar minute volume. If *V*_D-E_ is used for the optimal frequency calculation of ASV and AVM2, this mismatch will not occur, and AVM2 will have the lowest inspiratory power compared to ASV and MFV.

Given the assumption that *V*_D_ = 2.2 mL/kg, the initial settings using these targeting schemes will likely result in hypoventilation when actually ventilating patients with increased *V*_D_ (like ARDS and COPD). If, on the other hand, a factor based on measured physiologic *V*_*D*_ is used, then there is a greater likelihood that the initial result will be within the target range for CO_2_. The main point of interest for clinicians relates to modes that require manual adjustment of the minute ventilation target (e.g., all conventional modes, ASV, and AVM1). For these modes, underestimating the required minute alveolar ventilation by use of an estimate of *V*_D_ that is too small when making initial ventilator settings (frequency and tidal volume) will result in subsequent manual adjustments and delay in achieving the PaCO_2_ target. For modes that automatically adjust the minute ventilation using ongoing monitoring of etPCO_2_, subsequent adjustments are probably not as important an issue, but may still delay achievement of the desired PaCO_2_.

Nevertheless, the simulation reflects the practical experience as realistically as possible because we have the same mismatch in reality. We believe that optimal targeting schemes might be more “optimal,” in terms of their optimization target, if they used *V*_D-B_ instead of *V*_D-A_ or *V*_D-E_. Otherwise, they have a mismatch between the model (*V*_T_ = *V*_A_ + *V*_D_) and reality. On the other hand, there is clinical evidence that these targeting schemes work, with a few exceptions, for most lung conditions. If they are applied and understood correctly in practice, they may simplify clinical practice.

### Optimal ventilation and VILI

In the first section of this work, we analyzed analytically how ventilation would look like if the clinical indicators tidal volume, tidal pressure, tidal power, or driving power were stringently optimized. For tidal volume and tidal pressure, the solution would be in the range of *V*_*T*_~*V*_*D*_ at the highest possible frequency. At least for the ventilation of adults, this value seems to be far beyond the usual (green area in the figures). Also for the condition of minimal tidal power and driving power (*V*_*T*_ = 2·*V*_*D*_), *V*_*T*_ seems too low for normal lungs. But this does not necessarily mean that they are bad predictors for VILI. On the other hand, we observe from Figs. 3 and 4 that the inspiratory power for the adult simulations is more than a decade higher than that of the neonatal scenarios. As Marini already observed, adjustment to the reduced baby lung capacity may be necessary. This indicates that the cost function, which describes optimal ventilation, does not have to be necessarily a good predictor for VILI. Therefore, the answer to the questions “What should we optimize?” and “What induces lung injury?” might be not the same. However, it seems reasonable to understand that a cost function describing optimal ventilation should approach, at least for patients at risk for lung injury, the cost function that describes VILI.

We see from the simulation that the results are supporting Marini’s proposal to adjust an indicator for VILI for compliance (as mentioned above). This concept also seems to be confirmed (at least in a mathematical sense) by the fact that tidal pressure (driving pressure) can be interpreted as a compliance adjusted tidal volume. Finally, the comparison between neonatal and adult scenarios suggests that tidal pressure is more related to the lung conditions than to the weight of the patient.

### Limitations

For the simulations, we applied some simplifying assumptions and we did not include specific details reflecting the actual ventilator mode implementations of the targeting schemes. For example, ASV and AVM2 each have multiple “expert rules,” which could limit the frequency or tidal volume in certain scenarios (e.g., to avoid large tidal volumes or high intrinsic PEEP). Also, ASV is currently not designed for neonatal ventilation and limits its frequency to between 5 and 60 breaths per minute. Furthermore, AVM2 actually assumes an *I*:*E* ratio of 1:1.8, not 1:1, which was empirically derived during design of the mode implementation. This would lead to higher tidal volumes for normal lung conditions. MFV is designed to allow accommodation of unequal inspiratory and expiratory airway resistances.

## Conclusions

Modes of mechanical ventilation have shown a steady evolution over the last four decades. They have increased in complexity as engineers attempt to add technical capabilities that better serve clinical goals. A key feature of this complexity is the development of new targeting schemes, moving away from simple set-point targeting (all targets are operator preset) through adaptive targeting (some targets are automatically adjusted) to optimal targeting (targets are automatically adjusted to maximize or minimize some desired performance characteristic) and even intelligent targeting (automatic adjustment and selection of targets using the tools of artificial intelligence). In particular, optimum targeting schemes have been the central feedback control mechanisms of the most complex modes currently available. Optimization means, by definition, that there exists no better alternative to get, do, or set something, given the constraints of the mathematical model used. But we have shown that this kind of targeting scheme for ventilator modes is based on fairly arbitrary assumptions and presupposes clearly defined goals and targets, which are still topics of clinical debate. This paper tries to clarify these assumptions and point out that thinking about *what* should be optimized is much more important than thinking about *how* we should optimize. We suggest that optimization based on tidal volume, tidal pressure, or tidal power as the sole criteria may result in unusual ventilation strategies and settings. Therefore, we can adumbrate that modes based only on one of these variables may have limited clinical success.

### Additional information

All authors have approved this submission. The content of this manuscript has not been published, or submitted for publication elsewhere.

## Additional file

Additional file 1: Supplementary material. (DOCX 1009 kb)

## Abbreviations

$$ \widehat{\dot{V}} $$: Peak flow
$$ {\dot{W}}_{\mathrm{E}} $$: Elastic power
$$ {\dot{W}}_{\mathrm{insp}} $$: Inspiratory power
${\dot{W}}_{\mathrm{mus}}$: Breathing power (muscular power)
$$ {\dot{W}}_{\mathrm{PEEP}} $$: PEEP power
$$ {\dot{W}}_{\mathrm{R}} $$: Resistive power
$$ {\dot{W}}_{\mathrm{T}} $$: Tidal power
$$ \dot{V} $$: Flow
ASV: Adaptive support ventilation
AVM: Adaptive ventilation mode
C: Compliance
D: Fraction of the inspiration time Ti to the period T
*f*: Frequency (in Hz)
*f*_BF_: Frequency at minimal breathing force
*f*_BP_: Frequency at minimal breathing power
f_IP_: Frequency at minimal inspiratory power
f_TP_: Frequency at minimal tidal power
MFV: MID-frequency ventilation
MV: Minute volume measured at the proximal airway
MV_A_: Alveolar minute volume
MV_A-E_: Alveolar minute volume, calculated with the Enghoff modification of the Bohr equation
*P*_insp_: Inspiratory pressure
*P*_mus_: Pressure generated by inspiratory muscles
*P*_plat_: Plateau pressure
*P*_T_: Tidal pressure (aka driving pressure)
*R*: Airway resistance (linear)
*R*′: Airway resistance (non-linear portion)
RC: Time constant (product of resistance and compliance)
*V*: Volume
*V*_D_: Dead space volume
*V*_D-A_: Anatomical dead space
*V*_D-B_: Physiological dead space, calculated with the Bohr equation
*V*_D-E_: Physiological dead space, calculated with the Enghoff modification of the Bohr equation
*V*_T_: Tidal volume
