# Supplementary material for: Advanced modes of mechanical ventilation and optimal targeting schemes
Source: Intensive Care Med Exp. 2018 Aug 22;6:30. doi: 10.1186/s40635-018-0195-0 (PMC6104409; doi:10.1186/s40635-018-0195-0)
Supplement: Supplementary file 1 — Supplementary material. (DOCX 1009 kb) [file 40635_2018_195_MOESM1_ESM.docx]

# Advanced Modes of Mechanical Ventilation and Optimal Targeting Schemes

# Supplemental Material

**Authors**

Matthias van der Staay, MSc FHO in Engineering^1^

Robert L. Chatburn, MHHS, RRT-NPS, FAARC^2^

**Institutions**

1. imtmedical AG, Switzerland
2. Respiratory Institute, Cleveland Clinic, Cleveland, OH

**Conflict of Interest Statement**

- Robert Chatburn is a consultant for
  - imtmedical AG, Switzerland
  - IngMar Medical Inc., Pittsburgh, USA
  - Drive DeVilbiss Healthcare, Port Washington, NY, USA
- Matthias van der Staay is an employee of imtmedical AG

**Contributions**

Both authors participated in writing and review of the manuscript

# Abstract

Recent research results provide new incentives to recognize and prevent ventilator induced lung injury (VILI) and create targeting schemes for new modes of mechanical ventilation. For example, minimization of breathing power, inspiratory power, and inspiratory pressure are the underlying goals of optimum targeting schemes used in the modes called Adaptive Support Ventilation (ASV), Adaptive Ventilation Mode 2 (AVM2) and MID-Frequency Ventilation (MFV). We describe the mathematical models underlying these targeting schemes and present theoretical analyses for minimizing tidal volume, tidal pressure (also known as driving pressure) or tidal power as functions of ventilatory frequency. To go beyond theoretical equations, these targeting schemes were compared in terms of expected tidal volumes using different patient models. Results indicate that at the same ventilation efficiency (same PaCO_2_ level), we expect tidal volume dosage in the range of 7.4 mL/kg (for ASV), 6.2 mL/kg (for AVM2) and 6.7 mL/kg (for MFV) for adult ARDS simulation. For a neonatal RDS model, we expect 5.5 mL/kg (for ASV), 4.6 mL/kg (for AVM2) and 4.5 (for MFV).

# Key Words

mechanical ventilation, mathematical modelling, lung protective ventilation, optimal targeting schemes, simulation

# Introduction

The invention of the integrated circuit in 1958 has played an immense role in technical achievements during the last century. According to Moore’s law[1], the amount of transistors in an integrated circuit doubles every two years, implying that computing power of microprocessors has increased exponentially since 1958. In the last 60 years, this law has been fulfilled, and microprocessors have invaded every aspect of our lives. Today’s mechanical ventilators may use several microprocessors that enable them to deal with complex mathematical models as the basis for advanced modes of ventilation. By “mode of ventilation” we mean a predetermined interaction between patient and ventilator that can be characterized in terms of a control variable, a breath sequence, and one or more targeting schemes.[2] By “advanced” we mean that the mode makes use of software that gives the mode capabilities beyond simple feedback control of pressure or flow[3] (ie, “basic” modes). Taking this further, we could call some modes “adaptive”, meaning that they are advanced and also have the technical capability to serve at least two of the three goals of ventilation (ie, safety, comfort, and liberation). For example, simple volume control serves the goal of safety (assured tidal volume), simple pressure control serves the goal of comfort (ie, unrestricted inspiratory flow), but using adaptive targeting, a mode like Pressure Regulated Volume Control attempts to serve both goals (ie, unrestricted inspiratory flow and automatic adjustment of inspiratory pressure target to achieve an average preset tidal volume).

Advanced modes are often cascaded closed loop control systems, where the inner loop controls pressure and volume according to the equation of motion within a breath and the outer loop controls the rest of the mode between breaths. More specifically, technical advances have made possible many different “targeting schemes”[4] that result in the wide variety of modes of ventilation[2] seen on current ICU ventilators. In particular, the trend[5] in creating advanced modes includes also what are called “optimum” targeting systems.[4] The term optimum in this context means that a predefined mathematical criterion is specified as serving a goal of ventilation and its value is either minimized or maximized by automatically adjusting the ventilatory pattern in response to changes in the patient’s condition, as measured by mechanics and other physiologic signals (eg SpO_2_ and end tidal CO_2_).

From the perspective of an average clinician, optimal targeting schemes may appear to be “black magic”. This attitude can prompt either admiration and acceptance, or skepticism and rejection for use in routine ventilator management. The problem is that neither response is likely to be informed by facts and logic. To make matters worse, ventilator operator’s manuals often provide little explanation of how modes work (perhaps to protect intellectual property), and often the published research literature requires a high level of skill in physics, engineering, and mathematics, to understand. Therefore, the purpose of this review is to both explain the theory of optimum targeting systems and review the evidence supporting their use, as described in the current literature, in a way that will be useful for clinicians, educators, and researchers.

# What is a targeting scheme?

Basic feedback control within a breath, means that a target value (ie, inspiratory pressure, or volume and flow) is set by the user and a controller delivers gas to the patient in such a way that the difference between the set value and the instantaneous value of the feedback signal (measured or estimated pressure, or flow) converges to zero[3]. Specifically, the feedback values is subtracted from the set value to get an error value (negative feedback). If the set value is above the feedback value (ie, the error is negative), the controller will set the actuator in such a way that the feedback value (and hence, pressure or flow) will fall, and vice versa (

Figure 2).

Figure 2. Closed loop controller for basic pressure control mode. The pressure controller sets the actuators that make error (difference between measured patient pressure and set value) converge to zero.

As mentioned above, advanced ventilation modes typically have more than one control loop. These are usually cascaded control systems, where cascaded means, that an outer control loop (managing the ventilatory pattern) provides the set value for the inner control loop (managing pressure, volume, and flow as functions of time). These two control loops must be well coordinated. Common implementations of cascaded control loops are Autoflow (Dräger), Pressure Regulated Volume Control (Maquet), Volume Control Plus (Medtronics), Adaptive Pressure Ventilation (Hamilton), and Target Vent (imtmedical).

Figure 3. Advanced ventilation mode with cascaded control structure. The inner loop controls to pressure within breaths and the outer loop controls average volume over several breaths.

Adaptive ventilation modes are designed to automate some of the basic actions of clinicians as they attempt to optimize settings. The algorithms usually adapt to the changing characteristics of the patient such as mechanics (resistance, compliance, and inspiratory effort) or ventilatory pattern (frequency and tidal volume) and choose an “appropriate” response; but this also means that the machine has to know what is appropriate. One strategy to design clinical knowledge into the machine is called an optimum targeting scheme[2]. To create an optimum targeting scheme we need a model to mathematically describe “good” ventilation. In optimization theory, that model is also called a “cost function”. This function tells the machine how much a ventilation pattern “costs” in terms of predefined criteria, based on actual patient characteristics (e.g. the cost function could simply describe tidal volume dosage). After that, the task of the machine is to find the ventilation pattern with the lowest costs (optimum ventilation pattern). If this ventilation pattern is found, it can be used to set values (targets) for the underlying controllers

In 1991, Tehrani suggested that a “good” ventilatory pattern is one that mimics natural ventilation, and patented a targeting scheme based on the equation of Otis. She believed that this would “…reduce the load on the respiratory muscles, mimic natural breathing, stimulate spontaneous breathing, and reduce weaning time[6]. Otis et al. assumed that humans control their natural ventilation pattern “to the principle of minimum effort to which so many of the body functions seem to be regulated.”[7]. Accordingly, the cost function of Tehrani’s targeting scheme is the breathing effort (technically, average breathing power, or work rate) which has to be minimized. That targeting scheme was later commercialized by Hamilton Medical under the mode named Adaptive Support Ventilation (ASV) and by imtmedical under the mode named Adaptive Ventilation Mode (AVM).

Figure 4. Adaptive optimal targeting schemes like ASV (Adaptive Support Ventilation) and AVM (Adaptive Ventilation Mode). The system was designed to “…reduce the load on the respiratory muscles, mimic natural breathing, stimulate spontaneous breathing, and reduce weaning time”[6]. MV = minute ventilation, V_T_ = tidal volume, Paw = airway (transrespiratorysystem) pressure, RC = time constant as a product of resistance and compliance, f_opt_ = optimum frequency (minimal breathing power)

By serendipity, adopting a breathing pattern that minimizes breathing power of an unassisted patient leads also to a kind of optimizing power from the ventilator to the respiratory system. Hence the consequence is to maintain tidal volume dosage in a “lung protective range”, consistent with the current standard of care (ie, 6-8 mL/kg ideal body weight) that was established on the basis of studies conducted long after ASV was invented. Furthermore, minimizing power delivery may, in itself, contribute to a lung protective ventilation strategy[8, 9].

In 2013 Mireles-Cabodevila and Chatburn patented a new optimal targeting scheme called Mid Frequency Ventilation, a special application of pressure controlled intermittent mandatory ventilation.[10] Original descriptions of this mode emphasized selecting the ventilatory frequency that maximizes alveolar ventilation under the assumption of a predefined inspiratory pressure, and a later human study illustrated the modification of minimizing the tidal volume (by increasing frequency) for a required minute ventilation[11].

In 2017, imtmedical announced a modification of AVM called AVM 2[12]. This targeting scheme optimizes (ie, minimizes) inspiratory power (which is not the same as breathing power) and is designed to prevent ventilator induced lung injury (VILI). The idea behind AVM 2 is not to mimic spontenious breathing for passive patients. Instead the power which is delivered and remains in the patient is minimized. However, there are no clinical evaluations available yet.

In the first section of this paper, familiar ventilation parameters (tidal volume, tidal pressure, and tidal power) are used to derive cost functions. Also the cost functions, which underly the ventilation modes called Adaptive Support Ventilation (ASV), Adaptive Ventilation Mode 2 (AVM2) and Mid Frequency Ventilation (MFV), are derived. Next, we perform mathematical analyses to compare the characteristics of these optimum target schemes. These analyses tend to be complex and hard to interpret intuitively. For this reason, a summary of clinical evidence is presented along with mathematical simulations we performed to compare and visualize the results of the cost function minimization.

Figure 1 shows the variables used to derive the cost functions and how cost functions are used to optimize the ventilatory pattern.

Figure 1: Variables used for cost functions and targets. Every cost function (tidal volume, tidal pressure, tidal power, breathing power, inspiratory power and inspiratory pressure) was minimized analytically or using mathematical simulation. The result is the “optimal” ventilation frequency, which reaches the set alveolar minute ventilation and minimizes the cost function. After that, the frequency was used to calculate the target tidal volume.


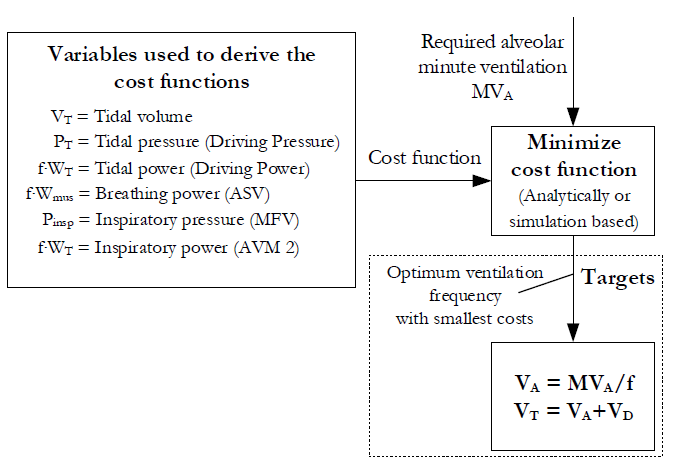


# A Closer Look at Optimum Targeting Schemes

## Basic engineering concepts to treat optimality

Usually there are several different solutions to solve a particular problem or different ways to achieve a goal. In order to achieve the best solution or the best way, researchers have developed different strategies, such as evaluation matrices, as well as the comparison of advantages and disadvantages. However, if we want to find optimality in a mathematical sense, we need to have a model (cost function) and make some assumptions about the model. As an example, if we want to calculate the most fuel efficient speed of a car, we need a mathematical model for fuel efficiency. To keep it simple, let’s assume that the fuel consumption is proportional to the power which is used for moving the vehicle, and that the needed power (for a given velocity) is only depended on the aerodynamic drag and the engine speed at idle. Under these assumptions, the fuel consumption can be approximated as:

|  | $\text{Fuel consumption}=c\cdot v^{3}+ b$ | (1) |
| --- | --- | --- |

where *v* is the velocity (car speed) and c is a coefficient which is depended on the car specific drag coefficient and density of the air. The constant b stands for the the idle fuel consumption (ie, when *v* = 0). Furthermore, we define fuel inefficiency (cost) as the fuel consumption per distance:

|  | $\text{Inefficiency}=\frac{\text{Fuel consumption}}{\text{Distance}}=\frac{c\cdot v^{3}+ b}{\text{v∙t}}$ | (2) |
| --- | --- | --- |

where distance can be expressed as the product of time and velocity. We now define optimum velocity as that which results in the lowest inefficiency. Therefore , we have to find the value of *v* that results in the lowest value of the cost function Equation (2):

|  | $\underset{v\in[0,\infty]}{arg min} \frac{c\cdot v^{3}+ b}{\text{v∙t}}$ | (3) |
| --- | --- | --- |

From this equation we get the value of the argument *v* in the interval between 0 and infinity that minimizes the cost function of inefficiency. Note that we are not interested in calculating the actual value for inefficiency at its minimum value, just the value of the associated velocity. For this reason we can simplify Equation (3) by neglecting the time, *t,* because that will not influence the optimal value for velocity. Hence, optimum velocity can be found by:

|  | $\underset{v\in[0,\infty]}{arg min} \frac{c\cdot v^{3}+ b}{\text{v}}$ | (4) |
| --- | --- | --- |

This optimization problem can be solved experimentally, numerically, or analytically. Experimentally means just trying out different values for *v* and calculate the cost function to find the location of the minimum. Numerically derived solutions are, for example, so called “fixed point iterations” whose final values will converge to a local minimum, depended on their initial condition (see Tehrani’s equation as an example[6]). We call a solution analytically derived when a optimization problem like Equation (4) can be solved directly (eg, through differentiation using calculus). Which of these approaches is used in practice depends primarily on the cost function, limitations relating to computing power, and requirements relating to the accuracy of the result.

To solve the problem of equation (4) experimentally, we randomly assume c = 0.1 and b = 10 and plot the cost function in terms of velocity *v*. Hence, we are able to visually assess its minimum at *v* ≈ 3.7.

Figure 5. Graph of the cost function in terms of velocity. The drag coefficient is assumed to be 0.1 and the bias 10. Due to visual assessment we identify the minimum value at velocity ≈ 3.7 where the slope (= first derivative) of the cost function becomes zero.


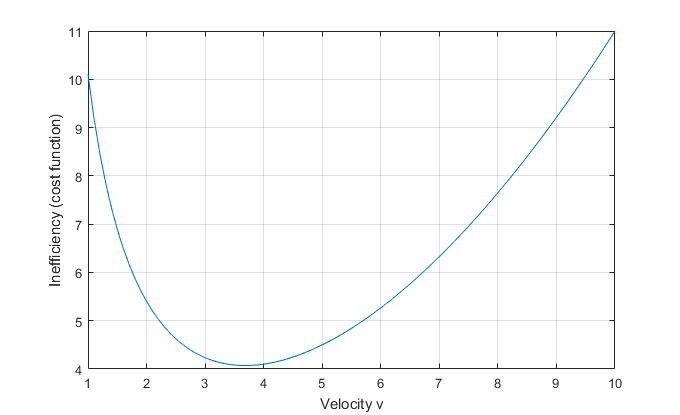


To derive the solution analytically, we can differentiate the cost function in respect to *v*. The resulting function can be interpreted as the slope of the cost function in terms of *v*. We can see in Figure 4, that the slope becomes 0 when the function reaches its minimum. For this reason we set the derivative to zero:

|  | $\left( \frac{c\cdot v^{3}+ b}{\text{v}} \right)\frac{d}{dv}=\frac{2\cdot c\cdot v^{3}-b}{v^{2}}=0$ | (5) |
| --- | --- | --- |

Solving this equation for *v* leads to:

|  | $v=\left( \frac{b}{2\cdot c} \right)^{1/3}$ | (6) |
| --- | --- | --- |

Hence, we are able to directly calculate the optimum velocity that will result in the lowest inefficiency (ie, highest fuel efficiency). Again setting *c* = 0.1 and *b* = 10, we get the exact solution *v* = 3.68.

Now we are ready to invent an optimal target scheme that automatically controls the speed of a car such that its fuel consumption per distance is minimized (and thus, for example, miles per gallon is maximized). To make the prediction of fuel consumption as precise as possible, the coefficient *c* could be estimated during driving (it was just assumed for this example). After the estimation of *c*, a target value for the velocity is calculated by equation (6), which in turn is used for the closed loop cruise controller.

Figure 6. Optimum targeting scheme for automatic cruise control. The velocity is automatically set in such a way that the car consumes as little fuel as possible. The parameter c is continually adapted to be as precise as possible.


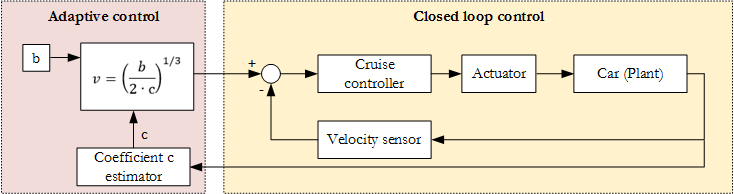


However, the optimal targeting scheme of Figure 5 works only to the extent that the cost function (or model) of equation (1) matches reality. In the car example, this is quite certainly not the case because many factors like tire and rolling resistance as well as friction in the car’s components and the characteristics of the engine are neglected. Hence, this simple targeting scheme will not find the “true” optimum and therefore, the fuel comsumption will not be minimized in reality.

The principles for an optimal targeting scheme as used for advanced ventilation modes are exactly the same. Instead of a model for fuel consumption, a cost function will be used for ventilation power or ventilation pressure. And instead of using a coefficient *c* estimator, ASV and AVM use the estimated time constant of the respiratory system (ie, the product of resistance and compliance). The cruise controller is replaced by a cascaded pressure/volume controller and the bias b can be replaced by the set value of the minute volume (ie, percent of predicted required minute ventilation).

As we can see, the notion of “optimum” depends strongly on the model, including targeting schemes as used for adaptive ventilation modes. We must also consider which simplifications are permissible and which are not in creating the model. Crooke and Marini have made important contributions to building models for mechanical ventilation. Crooke et al. presented a two-compartment model[13] and a model for nonpassive mechanical ventilation.[14] Marini et al. modeled and investigated the influence of ventilator settings in terms of tidal volume, minute volume, intrinsic PEEP, mean alveolar pressure and power for a one compartment lung model[15, 16].

# What should we optimize?

## Tidal Volume

The pivotal study by the Acute Respiratory Distress Syndrome Network in 2000 established the notion that in patients with acute lung injury and the acute respiratory distress syndrome, mechanical ventilation with a lower tidal volume dosage (6.2 vs 11.8 mL/kg ideal body weight) decreases mortality and increases the number of ventilator free days.[17] There are also data to support the use of low V_T_ in patients without pre-existing lung injury.[18–21] A recent study even suggests that lung protective ventilation might be considered a prophylactic therapy, rather than just a supportive therapy.[22]

If we assume a required alveolar minute volume ($\dot{V}_{A}$) and simply desire to control the tidal volume (*V_T_*) dosage for a passive patient, we can derive the cost function as follows:

|  | $V_{T}=\frac{\dot{V}_{A}}{f}+V_{D}$ | (7) |
| --- | --- | --- |

where V_D_ represents the dead space volume and *f* the ventilator frequency and hence $\frac{\dot{V}_{A}}{f}$ represents the alveolar volume. Thus, the “cost” in terms of tidal volume dosage (and presumably the risk of VILI) goes down as frequency goes up for a given required minute alveolar ventilation. Furthermore, if we set $\dot{V}_{A}$ = 5 L/min and V_D_ = 150 mL (reasonable values for a normal adult[23]) then we might be able to graphically evaluate where V_T_ is at the minimum value (assuming that minimal tidal volume is the desired goal to reach). However, we see in Figure 6 that there is no definite minimum value because tidal volume converges to the dead space volume as frequency increases to infinity. In practice, the limit would be dependent on the volume delivery performance characteristics of the ventilator, because no ventilator is a perfect flow controller. Also, in the United States, conventional ventilator frequency is limited to a maximum of 150 breaths/minute.

Figure 7. Tidal volume as a function of respiratory rate under the condition of a set alveolar minute volume of 5 L/min and dead space volume of 150 ml. It can be seen visually that the rate will converge to infinity for minimal tidal volume.


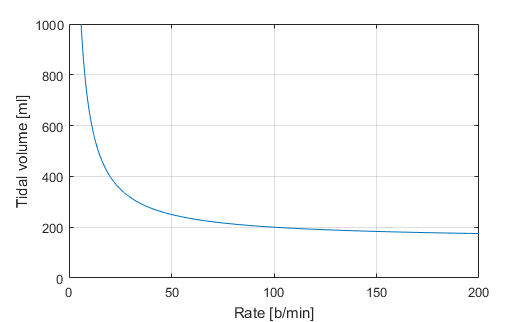


## Tidal Pressure

Simply controlling the tidal volume dosage, independent of any consideration of lung mechanics, may have limited utility. Recent work has suggested that V_T_ normalized to lung mechanics (eg, V_T_/C ), is a better predictor of mortality than tidal volume dosage.[24–26] We prefer to call V_T_/C (or equivalently, Pplt – totalPEEP) tidal pressure, P_T_, instead of driving pressure because P_T_ differs from V_T_ by only a scaling factor and driving pressure is sometimes used in reference to any pressure driving flow, not just static end-inspiratory pressure at the airway opening. In a cohort of brain-injured patients, P_T_ was associated with the development of ARDS.[27] In a series of ARDS patients receiving ECMO for refractory hypoxemia, P_T_ during ECMO was the only ventilator setting that showed an independent association with in-hospital mortality.[28] In patients having surgery, intra-operative high P_T_ and changes in the level of PEEP that resulted in an increase of P_T_ were associated with more postoperative pulmonary complications.[29] On the other hand, if V_T_ is strictly maintained at 6 mL/kg predicted body weight and P_plat_ below 28–30 cm H_2_O, then P_T_ shares the same information as P_plat_ about the association with day 90-mortality.[30]

However, if we would optimize to minimal P_T_, we get the same results as minimizing to tidal volume because the tidal pressure is linked to driving pressure by compliance, C, which can be considered simply a scaling factor. If compliance only affects the scaling of the cost function, then it has no influence to the location of the minimum.

|  | $P_{T}=\frac{1}{C}\cdot\left( \frac{\dot{V}_{A}}{f}+V_{D} \right)$ | (8) |
| --- | --- | --- |

## Tidal Power

Gattinoni et al have suggested an association between power transfer (from ventilator to lungs) and VILI.[8] However, as Marini and Jaber have observed[9] “…it is difficult to link power dissipated in proximal airway resistance directly to noxious events at the alveolar level.” Furthermore, they discount the effect of PEEP on the power equation because “…the ventilator’s work against PEEP is temporarily stored as potential energy within the elastic tissues of the respiratory system; it later converts to kinetic energy as the gas escapes to atmosphere across the exhalation valve.” Hence the power used to deliver the tidal volume against PEEP is not stored in the body and would not be expected to contribute to lung injury. Thus, they have suggested that a potentially better indicator of injury risk for clinical purposes might be “driving power” defined as:

|  | $\text{Driving Power}=\frac{f\cdot V_{T}\cdot P_{T}}{10\cdot C}$ | (3) |
| --- | --- | --- |

where C (compliance) is a scaling factor used to account for “…the reduced capacity of the ‘baby lung’”. However, we can define tidal power as

|  | $\text{Tidal Power}=\frac{f\cdot{V_{T}}^{2}}{2\cdot C}=\frac{f\cdot V_{T}\cdot P_{T}}{2}$ | (4) |
| --- | --- | --- |

which is equal to total power without the resistive portion and the energy which escapes to atmosphere during expiration. Marini and Jaber suggested driving power as a metric that could be associated with the risk of VILI and recommended that power be normalized “…at least for aerated lung capacity”. If tidal power is used as the cost function, we replace V_T_ in equation (4) with equation (1) and then solve the following optimization problem:

|  | $\underset{f \in[0,\infty]}{arg min} \frac{f}{2\cdot C}\cdot\left( \frac{\mathrm{MV}_{A}}{f}+V_{D} \right)^{2}$ | (5) |
| --- | --- | --- |

The solution is obtained analytically by differentiating tidal power with respect to f and setting the result to zero. Solving equation (5) for optimal frequency, leads to the remarkable result of

|  | $f_{\mathrm{TP}}=\frac{\text{MV}_{\text{A}}}{V_{D}}=\frac{\mathrm{MV}}{2\cdot V_{D}}$ | (6) |
| --- | --- | --- |

where f_TP_ = frequency of minimum tidal power, and MV = minute volume measured at the proximal airway. If we express MV as the product of tidal volume and frequency, the optimal tidal volume (ie, optimal in terms of minimal tidal power) can be expressed simply as function of dead space:

|  | $V_{T}=2\cdot V_{D}$ | (7) |
| --- | --- | --- |

Furthermore, if we assume V_D_ = 2.2 mL/kg (IBW) as an estimation for normal dead space volume, the tidal volume would be given by:

|  | $V_{T}=4.4 \text{mL/kg}$ | (8) |
| --- | --- | --- |

for the minimal tidal power to ventilate normal lungs. As mentioned, driving power is connected by a scaling factor to tidal power. Therefore, the condition of minimal driving power is fulfilled at the same optimal frequency and therefore yields the same optimal tidal volume.

Cressoni et al. defined transpulmonary mechanical work as the area between the inspiratory limb of the transpulmonary pressure vs volume curve during inspiration with constant flow[31]. They showed that if transpulmonary mechanical power (work per breath times respiratory frequency) exceeded the limit of 12 J/min, five out of five piglets developed whole-lung edema and four out of four did not when they were ventilated below that threshold.

The differences between tidal power (Equation (10)) and transpulmonary mechanical power are, that the compliance for tidal power is total respiratory system compliance (lungs plus chest wall) and the pressure does not include that due to inspiratory flow resistance and PEEP, whereas the compliance for transpulmonary power is lung compliance only and the pressure does include flow resistance and PEEP.

Interestingly, tidal power is currently not implemented by any ventilator in an optimum targeting scheme, although it is obvious that this would be feasible to do. But perhaps minimal power is not the ultimate goal for all patients across a range of different lung characteristics. It probably makes sense to consider, but automatically controlling ventilation on this sole criterion could conflict with other goals and objectives[4] (safety, comfort and liberation) of mechanical ventilation.

## Adaptive Support Ventilation to Minimize Breathing Power

In 1950, Otis et al. investigated unassisted breathing frequency with respect to lung mechanics and alveolar minute ventilation [32]. They made the assumption that the brain seeks an optimum frequency by minimizing breathing effort. To derive the cost function of breathing effort, they assumed a one compartment lung model with linear compliance and non-linear resistance:

|  | $P_{\text{mus}}=\frac{1}{C}\cdot V+R\cdot\dot{V}+R^{'}\cdot\dot{V}^{2}$ | (16) |
| --- | --- | --- |

where *R* is the linear (viscous) and *R’* the non-linear (turbulent) portion of airway resistance and *P_mus_* the pressure generated by inspiratory muscles. The flow $\dot{V}$ was assumed to follow a sine curve with an I:E ratio of 1:1:

|  | $\dot{V}(t)=\hat{\dot{V}}\cdot sin(2\cdot\pi\cdot f\cdot t)$ | (17) |
| --- | --- | --- |

where $\hat{\dot{V}}$ represents the peak flow. On the basis of that model, breathing effort was defined as work rate, or power. With the assumptions of equation (14) and (15), mean rate of muscular work was derived as[32]:

|  | $\underset{\text{Total Power}}{\underbrace{\dot{W}_{\text{mus}}}}=\underset{\text{Tidal Power}}{\underbrace{\frac{f}{2\cdot C}\cdot\left( \frac{\dot{V}_{A}}{f}+V_{D} \right)^{2}}}+\underset{\text{Resistive Power (viscous)}}{\underbrace{\frac{1}{4} \cdot R\cdot\pi^{2}\cdot\left( \dot{V}_{A}+f\cdot V_{D} \right)^{2}}} + \underset{\text{Resistive Power (turbulent)}}{\underbrace{\frac{2}{3}\cdot R'\cdot\pi^{2}\cdot\left( \dot{V}_{A}+f\cdot V_{D} \right)^{3}}}$ | (18) |
| --- | --- | --- |

To find the optimal frequency at minimal breathing power, the following optimization problem must be solved.

|  | $\underset{f\in[0,\infty]}{arg min} \dot{W}_{\text{mus}}$ | (19) |
| --- | --- | --- |

Figure 8. Total mean muscle power with its elastance and resistive components in terms of respiratory rate. With an alveolar minute volume = 6 L/min, R = 10 cmH_2_O/L/s, Rb = 5 cmH_2_O/(L/s)^2^, C = 50 mL/cmH_2_O and V_D_=150 mL the minimal muscular power can be located at a respiratory rate of approx. 15 bpm. That means, according to Otis’ assumption, that a human with the assumed lung mechanics would breath with 15 bpm when he needs 6 l/min alveolar minute volume.


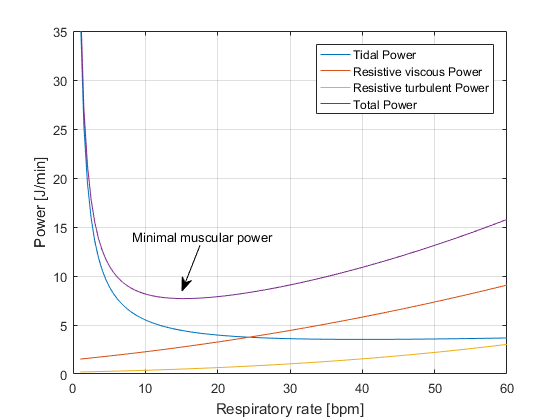


Otis solved equation (17) by differentiating equation (16) with respect to f and setting the result equal to zero. Instead of solving for *f,* he solved the equation for $\dot{V}_{A}$ to get a solution for the conditions of minimal breathing power (alveolar minute volume as a function of frequency). Later, Mead[33] simplified equation (14) neglecting the term with turbulent flow resistance ($R^{'}\cdot{\dot{V}(t)}^{2}$). Then he solved the optimization problem of (17), where the turbulent resistive power in equation (16) had disappeared through the simplification. This led to the well known equation for determining the optimal frequency at minimal breathing power (*f_BP_*):

|  | $f_{BP}=\frac{-1+\sqrt{1+\frac{4\cdot\pi^{2}\cdot RC\cdot\dot{V}_{A}}{V_{D}}}}{2\cdot\pi^{2}\cdot RC}$ | (20) |
| --- | --- | --- |

As an alternative to equation (18), Mead also showed that an optimal frequency exists at which the average force per breath required from the respiratory muscles is minimal (*f_BF_*):

|  | $f_{BF}=\left( \frac{\dot{V}_{A}}{V_{D}} \right)^{1/3}\cdot\left( 2\pi RC \right)^{-2/3}$ | (21) |
| --- | --- | --- |

Otis et al and Mead derived their equations to better understand the energetics of breathing and the associated effects on “the imaginary path from health to disease”. They were not concerned with inventing new modes of mechanical ventilation.

In 1991, Fleur T. Tehrani patented a targeting scheme based on Equation (20). The system was designed to “…reduce the load on the respiratory muscles, mimic natural breathing, stimulate spontaneous breathing, and reduce weaning time.”[6] Interestingly, the initial implementation of this targeting scheme was not to minimize power delivery from ventilator to patient,[34] but rather to select initial settings and “…choose a breathing pattern that encourages the patients to breathe on their own as early as possible.”[35] Note that the development of this targeting scheme was almost a decade before intensive research on the role of tidal volume dosage on mortality. At that time, the concern was to avoid an excessively large tidal volume, not to minimize it. Nevertheless, over the years, ASV has proven to be effective and results in relatively protective tidal volume delivery in the range of 8.1 ± 1.4 mL/kg ideal body weight.[36]

Note however, that the Otis and Mead equations assume a *sinusoidal* pressure waveform, whereas ASV was implemented with a *square* pressure waveform. Thus, for any given optimum frequency, a square pressure waveform (ie, constant inspiratory pressure) will yield a larger tidal volume than a sine wave with the same inspiratory pressure amplitude (for a square waveform the amplitude is PIP – PEEP and for a sine it is the peak value or P_max_). This is because tidal volume is the product of compliance and the mean inspiratory pressure. For a square pressure waveform, the mean pressure is the same as the amplitude, PIP-PEEP. But for sine pressure waveform, the mean inspiratory pressure is less; 0.637 × P_max_. Perhaps this is the reason that ASV delivers about 8 mL/kg rather than the desired lung protective value of about 6 mL/kg.[37]

## Adaptive Ventilation Mode 2 to Minimize Inspiratory Power

Ventilation modes using adaptive targeting based on Otis equation do not necessarily deliver lung protective ventilation.[38, 39] To reduce tidal volume (and subsequently tidal pressure),[40] we can derive the concept of mean inspiratory power.[12] Inspiratory power is defined as the sum of the resistive and tidal power which is transmitted from the ventilator to the patient, assuming intrinsicPEEP equal zero:

|  | $\text{Inspiratory Power (}\dot{W}_{\mathrm{insp}}\text{) = }\text{Tidal Power (}\dot{W}_{T}\text{)}\text{+Resistive Power (}\dot{W}_{R}\text{) }$ | (15) |
| --- | --- | --- |

There are differences among inspiratory power, total power[8], elastic power, breathing power and tidal power. Elastic power includes tidal power and PEEP power, inspiratory power includes tidal power and resisitve power, and total power includes elastic power and resistive power. Figure 9 and Table 1 explain these concepts (which were created by Otis, Gattinoni, Marini, and us). Note that power is defined as the work per unit time, which is calculated as the product of work and ventilatory frequency. Inspiratory work per breath is defined as the integral of inspiratory pressure with respect to inspiratory volume, or graphically, the area between the pressure curve and the volume axis as shown in Figure 9.

Figure 9: Definition of the different power components of an inspiration. Breathing power was introduced by Oti’s, resistive and elastic power was defined by Gattinoni, and Marini differentiated elastic power into its components PEEP power and tidal power. The autors now introduce the concept of inspiratory power which is composed of tidal and resistive power. Note, that the figure shows work instead of power and power is the result of the product between work and ventilation frequency.


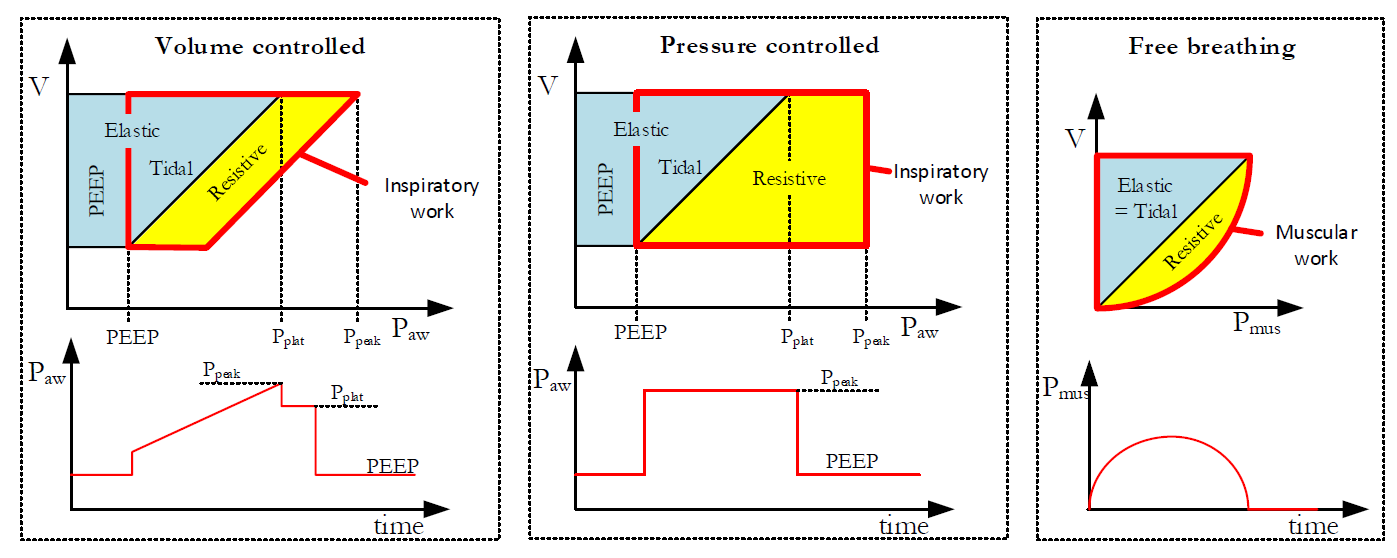


| Total Work: | Work generated by the ventilator to deliver pressure and flow to the respiratory system. |
| --- | --- |
| Resistive Work: | Work Energy which is dissipated (as heat) in in generating flow through the airway resistance . |
| Elastic Work: | Work needed to expand the lungs and chest wall. |
| PEEP Work: | Work against PEEP during inspiration which is temporarily stored as energy within the elastic tissues. It later converts to kinetic energy as the gas escapes to atmosphere across the exhalation valve. |
| Tidal Work: | Elastic Work minus PEEP |
| Inspiratory Work: | The sum of Tidal Work and Resistive Work. |
| Muscle Work: | Work generated by the ventilatory muscles. |

Table 1: Definition of the different energy portions relating to Figure 9.

There is an important difference between *muscle* power $\dot{W}_{\mathrm{mus}}$ and *inspiratory* power. Otis derived the mean power which is needed to breathe *without* the support of a ventilator with a *sinusoidal muscle pressure waveform*. On the contrary, the concept of inspiratory power relies on the principle of how much power is delivered to the patient by a *ventilator using a* *square pressure waveform* (assuming total PEEP equals zero).

Inspiratory power is not intended to be another predictor for VILI. Instead, it serves as the basis for defining an alternative cost function which may be used to describe an optimal ventilation pattern. Inspiratory power includes not only tidal power (which might be a better indicator for VILI) but also includes resistive power. This leads to a more “natural” ventilation similar to Otis’ breathing power. However, as we will see, minimizing inspiratory power converges for specific patient characteristics to the same result as minimizing tidal power which might be relevant for VILI prevention.

The inspiratory power can be calculated as the sum of tidal power and resistive power. Tidal power is given by formula (10) and resistive power for pressure controlled ventilation (with constant inspiratory pressure) can be calculated for a one compartment linear lung model by:

|  | $\begin{matrix} \dot{w}_{R} & =f\cdot\int_{0}^{T_{I}} R\cdot{\dot{V}(t)}^{2}dt \\ & =f\cdot\int_{0}^{T_{I}} R\cdot\left( \frac{V_{t}\cdot e^{-\frac{t}{R\cdot C}}}{R\cdot C-R\cdot C\cdot e^{-\frac{T_{I}}{R\cdot C}}} \right)^{2}dt \\ & =\frac{1}{2\cdot C}\cdot f\cdot{V_{T}}^{2}\cdot coth\left( \frac{T_{I}}{2\cdot R\cdot C} \right) \end{matrix}$ | (24) |
| --- | --- | --- |

where coth() is the mathematically defined cotangens hyperbolicus function and T_I_ the inspiratory time.

Hence, inspiratory power can be calculated as:

|  | $\dot{w}_{Insp}=\frac{1}{2\cdot C}\cdot f\cdot\left( \frac{\dot{V}_{A}}{f}+V_{D} \right)^{2}\cdot\left( 1+coth\left( \frac{T_{I}}{2\cdot R\cdot C} \right) \right)\text{ }$ | (25) |
| --- | --- | --- |

To find the frequency for minimal inspiratory power *f_IP_*, the following optimization problem must be solved.

|  | $\underset{f\in[0,\infty]}{arg min} \dot{w}_{Insp}$ | (26) |
| --- | --- | --- |

Assuming an I:E = 1:1, following numerical solution can be derived:

|  | $f_{IP}=\frac{\mathrm{MV}}{2\cdot V_{D}}\left( 1-\frac{1}{2\cdot f_{IP}\cdot R\cdot C\cdot\left( e^{\frac{1}{2\cdot f_{IP}\cdot R\cdot C}}-1 \right)} \right)$ | (27) |
| --- | --- | --- |

Note that equation (27) is a so called “fixed point iteration”. That means that we can not directly calculate the optimal frequency *f*_IP_. The optimum frequency is found in an iterative numerical process starting with a seed value. To understand and interpret equation (27) more intuitively, the equation can be also written as:

|  | $f_{IP}=\frac{\mathrm{MV}}{2\cdot V_{D}}\cdot K= f_{TP}\cdot K$ | (28) |
| --- | --- | --- |

where K is a factor which is depended on the product of the respiratory time constant RC and the frequency *f*. In Figure 8 it can be seen that the value of K is always between 0 and 1 if $\left( R\cdot C\cdot f \right)$is a positive real number. We can recognize that for K approaching 1, the solution of equation (27) converges to the solution for minimal tidal power of equation (13). For K approaching 0, the frequency converges to 0. Therefore *the* *optimal frequency for minimal inspiratory power is always equal or below the frequency for minimal tidal power*.

|  | $f_{IP}\leq f_{TP}$ | (29) |
| --- | --- | --- |

Futhermore it can be shown that K approaches 0 for high values of the time constant (RC) and vice versa; K approaches 1 for low time constants. It makes intuitive sense that respiratory rate decreases for slow time constants and increases for fast time constants, as this is how the brain normally responds to changes in lung mechanics as explained by Otis.

Figure 10. Relation between factor K and $\text{f}\text{ ∙R∙C}$. Note that both terms are unitless. K can be interpreted as the fraction between *f*_TP_ and *f*_IP_ which is always between 0 and 1. Hence, the frequency for minimal inspiratory power is always equal or below the frequency for minimal tidal power.


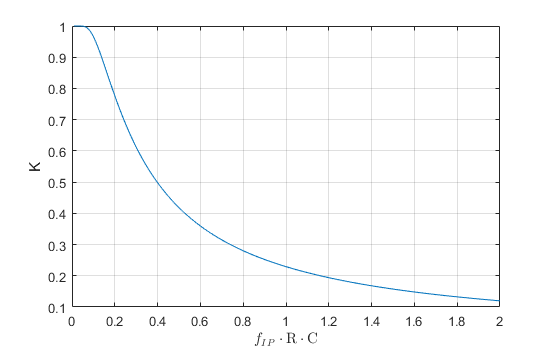


## Alveolar Ventilation or Inspiratory Pressure

Marini et al derived an equation which allows us to predict tidal volume in terms of ventilator settings and lung mechanics[16]. In 2013, Chatburn and Mireles-Cabodevila extended this equation to predict alveolar minute volume as a function of frequency, and invented a new optimal targeting scheme called Mid Frequency Ventilation (MFV)[10]. MFV is designed to maximize alveolar minute ventilation for a given inspiratory pressure target[10] or minimize inspiratory pressure target for a target minute alveolar ventilation[11] (inspiratory pressure is the preset pressure change above PEEP on the ventilator, ie, the amplitude of the square pressure waveform).

According to Marini, tidal volume can be expressed as:

|  | $V_{T}=P_{\text{insp}}\cdot C\cdot\frac{(1-e^{-\frac{D}{f\cdot R_{I}\cdot C}})\cdot(1-e^{-\frac{1-D}{f\cdot R_{E}\cdot C}})}{(1-e^{-\frac{D}{f\cdot R_{I}\cdot C}}\cdot e^{-\frac{1-D}{f\cdot R_{E}\cdot C}})}$ | (30) |
| --- | --- | --- |

where R_I_ is the inspiratory resistance, R_E_ the expiratory resistance and D the fraction of Ti to the period T. The ventilation frequency *f* is denoted in hertz. With a constant dead space volume V_D_, the alveolar minute volume is given by:

|  | $\begin{matrix} \dot{V}_{A} & =f\cdot(V_{T}-V_{D}) \\ & = f\cdot\left[ P_{\text{insp}}\cdot C\cdot\frac{\left( 1-e^{-\frac{D}{f\cdot R_{I}\cdot C}} \right)\cdot\left( 1-e^{-\frac{1-D}{f\cdot R_{E}\cdot C}} \right)}{\left( 1-e^{-\frac{D}{f\cdot R_{I}\cdot C}}\cdot e^{-\frac{1-D}{f\cdot R_{E}\cdot C}} \right)}-V_{D} \right] \end{matrix}$ | (31) |
| --- | --- | --- |

To get the optimal ventilation frequency, the following equation must be solved to maximize alveolar minute volume:

|  | $\underset{f\in[0,\infty]}{arg max} \dot{V}_{A}$ | (32) |
| --- | --- | --- |

This can be solved experimentally by trying out different frequencies[10]. Equation (31) can be also used in a different way to minimize inspiratory pressure under the condition of constant minute volume. Hence, it can be used in the same way as the equations of ASV or AVM 2. That means, that we are able to find the minimal required inspiratory pressure for a set required alveolar minute volume. The frequency of minimal inspiration pressure (P_insp_) can be found by solving following optimization problem:

|  | $\underset{f\in[0,\infty]}{arg min} P_{\text{insp}}$ | (33) |
| --- | --- | --- |

where P_insp_ can be expressed by rearranging Equation (31) as:

|  | $\begin{matrix} P_{\text{insp}} & =\underset{\text{Tidal pressure}}{\underbrace{\frac{\dot{V}_{A}+f\cdot V_{D}}{f\cdot C}}}\cdot\frac{\left( 1-e^{-\frac{D}{f\cdot R_{I}\cdot C}}\cdot e^{-\frac{1-D}{f\cdot R_{E}\cdot C}} \right)}{\left( 1-e^{-\frac{D}{f\cdot R_{I}\cdot C}} \right)\cdot\left( 1-e^{-\frac{1-D}{f\cdot R_{E}\cdot C}} \right)} \end{matrix}$ | (34) |
| --- | --- | --- |

Equation (33) can be also solved experimentally by trying out different values for ventilation frequency. It seems to be logical that the same frequency which maximizes alveolar minute volume will also minimizes inspiratory pressure if the same conditions are used (Figure 8).

Figure 11. Optimization on inspiratory pressure (blue) and alveolar minute volume (orange). With an alveolar minute volume = 6 L/min, R_I_ =R_E_ = 10 cmH2O/L/s, C = 30 ml/cmH2O, I:E = 1:1 and V_D_=150 mL the minimal P_Insp_ of 11.7 cmH2O can be located at a respiratory rate of approx. 44 bpm (left axis). Using P_Insp_ of 11.7 cmH2O for maximizing alveolar minute volume, will lead to an alveolar minute volume of 6 L/min at same respiratory rate of 44 bpm (right axis). The vertical black line is placed on the optimum rate.


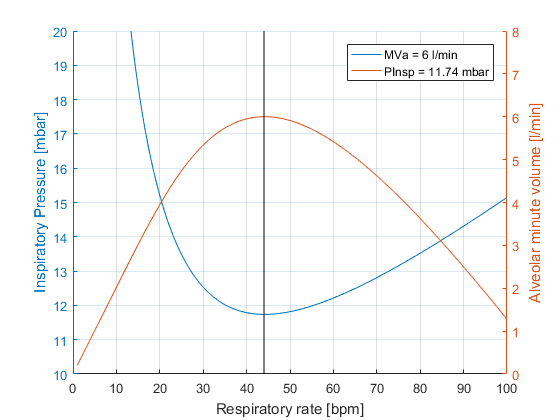


# Evidence for Optimum Targeting Schemes

In this section we report the scientific evidence for the three modes of ventilation using optimum targeting schemes as described above. However, any interpretation of clinical meaning of the evidence is beyond the scope of this article.

First, we point out that there are 3 different varieties of IMV.[2] The original, or Type I IMV was designed so that the operator could set a frequency for mandatory breaths (ie, those that are either triggered or cycled by the ventilator) and they would be delivered regardless of patient trigger activity. Type II was made popular by Respironics in the “Spontaneous/Timed” mode of PC-IMV where spontaneous breaths (ie, those that are both triggered and cycled by the patient) will suppress mandatory breaths when the spontaneous breath frequency exceeds the set mandatory breath frequency. Type III IMV originated with the invention of Mandatory Minute Ventilation (MMV) for which mandatory breaths are suppressed if spontaneous breaths keep the minute ventilation above the preset target value. Thus, ASV and AVM 2 are examples of Type III IMV while MFV (in its current experimental form) is Type I.

Other modes mentioned in the studies below, include PC-CMVs (pressure control continuous mandatory ventilation with set-point targeting, eg, Pressure Assist/Control), VC-CMVs (volume control continuous mandatory ventilation with set-point targeting, eg, Volume Assist/Control), PC-CSVs (pressure control continuous spontaneous ventilation with set-point targeting, eg, Pressure Support), PC-CMVa (pressure control continuous mandatory ventilation with adaptive targeting, eg, Pressure Regulated Volume Control), PC-IMVs,s (pressure control intermittent mandatory ventilation with set-point targeting for both mandatory and spontaneous breaths, eg, SIMV Pressure Control).

## ASV and IntelliVent ASV

Much has been written about ASV (note that IntelliVent ASV is an advanced variety of ASV, with the same taxonomic mode classification but with the addition of automatic control of minute ventilation target, PEEP, and FiO_2_). In preparing this manuscript, a Google search on “Adaptive Support Ventilation” revealed 72 references between 2000 and 2017. ASV evolved as a form of MMV that was described by Hewlett et al. in 1977[41]. Table 1 shows the basic research on ASV. Table 2 shows studies of ASV in animals and Table 3 shows the human studies.

Table 2: Basic research of ASV

| Author | Year | Study Purpose | Conclusions |  |
| --- | --- | --- | --- | --- |
| Laubscher | 1994 | Original description of ASV | The new targeting scheme was found to respond to step changes in model parameters within 48 to 81 seconds with overshoot between 5.5% and 7.9%. | [42] |
| Belliato | 2004 | Evaluated ASV using a lung simulator set with three lung models (normal, restrictive, and obstructive). Minute ventilation was set at 100% and 130% of predicted. | ASV acted as expected for ventilation of different lung categories. Furthermore, the selected pattern seemed to be adequate compared to settings proposed by a skilled operator with a conventional mode of ventilation. | [43] |
| Sulemanji | 2009 | Compared ASV to VC-CMVs during simulated ARDS | ASV was better able to prevent the potential damaging effects of excessive plateau pressure (greater than 28 cm H_2_O) than VC-CMVa with a fixed tidal volume of 6 mL/kg by automatically adjusting airway pressure, resulting in a decreased tidal volume. | [44] |
| Veelo | 2010 | Used a breathing simulator to analyze tidal volume delivery for different lung models (ie, combinations of resistance and compliance) representing normal, acute lung injury (ALI), and COPD. | For ALI tidal voluimes were 6-8 mL/kg; for normal lungs 8-10 mL/kg and for COPD all tidal volumes were above 10 mL/kg. | [45] |
| Morato | 2012 | Compared ASV with SmartCare and Mandatory Rate Ventilation. | All three modes were equally able to recognize weaning success and failure, despite the presence of anxiety or irregular breathing, but performed incorrectly in the presence of Cheyne-Stokes. | [46] |
| Mireles-Cabodevila | 2012 | Used a survey to determine clinician selected settings (during VC-CMVs) for various disease states (normal, ARDS, morbid obesity, COPD, and status asthmaticus). The survey-derived values for minute ventilation and minute alveolar ventilation were used as goals for ASV and MFV, respectively. A lung simulator programmed with each scenario's respiratory system characteristics was ventilated using the clinician, ASV, and MFVsettings. | Tidal volumes ranged from 6.1 to 8.3 mL/kg for the clinician, 6.7 to 11.9 mL/kg for ASV, and 3.5 to 9.9 mL/kg for MFV. Inspiratory pressures were lower for ASV and MFV. Clinician-selected tidal volumes were similar to the ASV settings for all scenarios except for asthma, in which the tidal volumes were larger for ASV and MFV. MFV delivered the same alveol arminute ventilation with higher end expiratory and lower end inspiratory volumes. | [47] |
| Sulemanji | 2013 | Compared VC-CMVs and PC-CMVs to ASV with and without closed loop control by end tidal CO_2_ using a simulator to model patients with normal lungs, ARDS, and COPD | All modes performed similarly in most cases with minor differences that favored closed loop control of CO_2_. | [48] |

Table 3: Animal studies of ASV

| Author | Year | Population | Study Purpose | Conclusions |  |
| --- | --- | --- | --- | --- | --- |
| Jung | 2010 | 12 piglets divided between two modes | Compared the effects of ASV with those of PC-CMVs on both in vivo and in vitro diaphragmatic properties of healthy piglets. | After 72 hours of ventilation, transdiaphragmatic pressure decreased by 30% of its baseline value in the PC-CMVs group, whereas it did not decrease in the ASV group. They concluded that ASV may protect the diaphragm against the deleterious effect of prolonged PC-CMVs. | [49] |

Table 4: Human studies of ASV and ALV. The prototype of ASV was called ALV (Adaptive Lung Ventilation)

| Author | Year | Population | Study Purpose | Conclusions |  |
| --- | --- | --- | --- | --- | --- |
| Laubscher, Heinrichs | 1994 | 6 adults - normal | Evaluated the initial settings selected by ALV | Well tolerated by all patients and performed as expected | [50] |
| Laubscher, Frutiger | 1994 | 25 adults, 17 children – critical ill | Evaluated the initial settings selected by ALV during 1 minute of "test breaths" | Initial settings proposed by ALV after test breaths was appropriate for 100% of patients > 3 years old and 75% < 3 years old | [35] |
| Weiler | 1994 | first series of ventilated patients | Tested ALV after a step change in minute ventilation | Breathing patterns determined by ALV were well adapted to lung mechanics. Respiratory rates, inspiratory pressures, and tidal volumes were within clinically acceptable range for all patients. | [51] |
| Linton | 1994 | 27 adults - normal, parenchymal lung disease, COPD | Evaluated ALV selected settings when weaning criteria are first met | Provided safe, efficient weaning and will respond immediately to inadequate ventilation | [52] |
| Laubscher | 1996 | 20 adults - during surgery in nephrectomy position | Evaluated ventilatory parameters selected by ALV and compared to VC-CMVs | The effects of positioning for nephrectomy are minor and may give rise to problems only in patients with restrictive lung disease. ALV automatically selected ventilatory parameters that were clinically sound and better adapted to the respiratory mechanics of ventilated patients than the standardized settings of conventional ventilation. | [34] |
| Weiler | 1998 | 9 adults - during anesthesia for pulmonary surgery | Investigated changes in respiratory mechanics during transition to and from one-lung ventilation and described ALV adaptation of ventilatory pattern | Transition to one-lung ventilation caused marked changes in resistance and compliance in opposite directions, leaving the time constant unaffected. ALV managed transition successfully, maintained minute alveolar ventilation and kept tidal volume lower than recommended | [53] |
| Sulzer | 2001 | 36 adults - post uncomplicated cardiac surgery | Tested the hypothesis that a protocol of respiratory weaning based on ASV could reduce the duration of tracheal intubation | ASV may accelerate tracheal extubation and simplify ventilatory management in fast-track patients | [54] |
| Tassaux | 2002 | 10 adults - acute respiratory failure | Compared VC-IMVs modes on patient-ventilator interactions in patients undergoing partial ventilatory support | In patients with signs of increased respiratory muscle loading, ASV provided levels of minute ventilation comparable to those of VC-IMVs. With ASV, central respiratory drive and sternocleidomastoid activity were markedly reduced. | [55] |
| Cassina | 2003 | 155 adults - post uncomplicated cardiac surgery | Evaluated ASV for fast-track weaning | This mode was safe, easy to apply, and allowed rapid extubation | [56] |
| Petter | 2003 | 36 adults - post uncomplicated cardiac surgery | Tested the capability of ASV to perform weaning automatically compared to VC-IMVs and PC-CSVs | Outcomes with ASV were similat to conventional ventilator management but with less manipulation of settings and alarms | [57] |
| Belliato | 2004 | 21 adults - normal, acute and chronic respiratory failure - sedated and paralyzed | Evaluation of respiratory pattern selected by ASV | In normal patients ASV selected a ventilatory pattern comparable with the physiological one. In the obstructive patients the pattern was low respiratory rates and high tidal volumes. In the restrictive patients the pattern was high respiratory rates and low tidal volumes and always within 6 mL/kg | [43] |
| Linton | 2006 | 27 adults - ventilator dependent | Evaluated ASV as sole mode in chronic care facility | Safe, effective, and half of the patients were successfully weaned between 2 weeks and 2 months. | [58] |
| Arnal | 2008 | 243 adults - normal, ARDS, COPD, acute respiratory failure | Evaluated ASV performance in passive and actively breathing patients with various categories of jung disease | ASV selected different tidal volume and rate combinations as expected based on mechanics in passive and active patients | [59] |
| Gruber | 2008 | 48 adults - post coronary artery bypass grafting surgery | Determined whether ventilation in ASV resulted in a shorter time to extubation than PC-CMVa | ASV was associated with earlier extubation, without an increase in clinician intervention | [60] |
| Dongelmans | 2008 | 262 adults - weaned after post-op cardiac surgery | Identification of factors that affect tidal volume | Tidal volume was dependent on respiratory rate and correctness of set body weight and was > 8 mL/kg in a substantial number of patients | [61] |
| Veelo | 2008 | 34 adults - percutaneous dilational tracheotomy with endoscopy | Determined need for changes in minute ventilation | For 74% of patients, ASV was unable to maintain minute ventilation during the use of the endoscope, mandating pressure limitation adjustments. In a minority of patients (26%), minute ventilation had to be adjusted to achieve similar PaCO2 values. | [62] |
| Dongelmans | 2009 | 128 adults - post cardiac artery bypass grafting surgery | Determined the effect of ASV on time to tracheal extubation in non-fast track patients | Weaning with ASV was safe. Time until extubation was no different from standard weaning. | [63] |
| Llorens | 2009 | 22 adult women - gynecological laparoscopic surgery | Tested efficacy of ASV to automatically adapt to changes in respiratory mechanics that occur during trendelenburg position | ASV automatically adapbed settings to respiratory mechanics keeping constant minute ventilation and adequate gas exchange | [64] |
| Jaber | 2009 | 14 adults - ICU patients being weaned | Evaluated increased respiratory demand, where the level of support may be decreased with PC-CMVa but not with ASV or PSV | ASV and PC-CSVs behaved differently but ended up with similar pressure level facing acute changes in ventilatory demand. In contrast, PC-CMVa results in a decrease in inspiratory pressure | [65] |
| Dongelmans | 2010 | 126 adults - non fast-track cardiothoracic surgery patients | Compared standard ASV and ASV with lower % minute ventilation to increase spontaneous breath rate and speed weaning | Decreased % minute ventilation setting does not shorten time to extubation | [66] |
| Veelo | 2010 | 119 adults - post coronary artery bypass grafting surgery | Determined tidal volume size | Tidal volume was between 7 and 9 mL/kg | [45] |
| Iotti | 2010 | 88 adults - normal, restrictive and obstructive lung disease | Compared ASV with VC-CMVs and PC-CMVs in terms of gas exchange, compare automatic with human choices for ventilator settings, and determined if ASV delivers larger tidal volumes | Comparison between ASV and conventional modes resulted either in similarities or in minor differences. Except for excessive tidal volume in a few obstructed patients, all differences were in favor of ASV | [67] |
| Dongelmans | 2011 | 10 adults - with acute lung injury | Tested hypothesis that ASV delivers higher tidal volumes than recommended | Adaptive support ventilation delivered a lower respiratory rate-higher tidal volume combination. Pressure limitation did correct for the rise of tidal volume but led to a decline in minute ventilation. | [68] |
| Chen | 2011 | 149 adults - medical ICU | Tested hypothes is that ASV would improve probability of ventilator liberation | Extubation readiness may not be recognized in a timely manner in at least 15% of patients recovering from respiratory failure. ASV helps to identify these patients and may improve their weaning outcomes. | [69] |
| Kirakli | 2011 | 97 adults - COPD during weaning | Tested hypothesis that weaning with ASV could reduce weaning duration | ASV provided shorter weaning times with similar weaning success rates | [70] |
| Arnal | 2012 | 50 adults - passive stable patients with moderate acute respiratory failure | Compared safety and efficacy of ASV vs IntelliVent ASV | IntelliVent-ASV was safe and able to ventilate patients with less pressure, volume, and FiO_2_ while producing the same oxygenation | [71] |
| Agarwal | 2013 | 48 adults - ARDS | Compared VC-CMSs to ASV | There was no significant difference in duration of ventilation, organ failure scores, ICU/hospital stay, or mortality | [72] |
| Celli | 2014 | 20 adults - post-op for orthotopic liver transplantation | Compared PC-IMVs to ASV | ASV was superior in terms of weaning times, and it simplified respiratory management | [73] |
| Han | 2014 | 86 adults - COPD | Compared effects on peripheral circulation | PaO_2_, pH, and SaO_2_ values were remarkably increased with ASV | [74] |
| Zhu | 2015 | 53 adults - post-op cardiac valvular surgery | Tested hypothesis that ASV would be associated with shorter duration of ventilation compared to VC-CMVs and VC-IMVs | ASV reduced ventilation time by more than 2 hours while reducing the number of manual ventilator changes and alarms | [75] |
| Kirakli | 2015 | 229 adults - medical ICU | Compared duration of mechanical ventilation to PC-CMVs | ASV may shorten the duration of weaning and total duration of ventilation with a fewer number of manual ventilator settings | [76] |
| Yazdannik | 2016 | 64 adults - post-op coronary artery bypass graft surgery | Compared duration of mechanical ventilatino and hospital stay to VC-IMVs | ASV led to a decrease in intubation duration and also hospital stay | [77] |
| Tam | 2016 | 52 adults - post-op cardiac surgery | Compared a protocol using a decremental minute ventilation target compared to a protocol with constant minute ventilation target | Use of a decremental protocol resulted in a shorter duration of ventilation and intubation without evidence of increased risk of adverse effects | [78] |
| Ghodrati | 2016 | 60 adults - neurosurgical ICU | Compared respiratory parameters to VC-IMVs | ASV mode can lead to improved lung compliance and respiratory dead space | [79] |
| Moradian | 2017 | 115 adults - post-op cardiac surgery | Compared VC-IMVs and PC-CSVs for effects on atelectasis | ASV could reduce the incidence of atelectasis and length of hospital stay. However, it did not reduce the duration of mechanical ventilation. It seems that ASV is not a superior mode for faster extubation. | [80] |
| Kaiei | 2017 | 40 adults - ICU patients | Compared ASV with minute ventilation targets of 110% vs 120% | Using ASV with target minute ventilation of 120% can decrease extubation time compared with target of 110%. Furthermore, there was not a considerable side effect on hemodynamic of patients. | [81] |
| Peng | 2017 | 337 adults - acute respiratory failure | The authors previously showed that for patients receiving ASV there existed a Transition %MinVol (TMV%) where the patient's work of breathing began to reduce. In this study, they tested the hypothesis that higherTMV%would be associatedwith poorer outcomein patients with acute respiratory failure. | An increase, or lack of decrease, of TMV% from Day 1 to Day 2 was associated with higher risk of in-hospital death | [82] |

## Mid-Frequency Ventilation, MFV

Mireles-Cabodevila and Chatburn[10] introduced MFV in 2008. They used an interactive mathematical model of ventilator output during pressure control ventilation (ie, MFV) to predict the frequency at which alveolar ventilation is maximized with the lowest tidal volume for a given inspiratory pressure. Table 4 shows basic research on MFV. Table 5 shows the single animal study and Table 6 shows the single human study of MFV.

Table 4: Basic research of MFV

| Author | Year | Study Purpose | Conclusions |  |
| --- | --- | --- | --- | --- |
| Mireles-Cabodevila | 2008 | Tested the predicted optimum frequency and tidal volume values with various passive lung models (ie, different resistances and compliances) with a simulator connected to five diferent ventilators. | After 72 hours of ventilation, transdiaphragmatic pressure decreased by 30% of its baseline value in the PC-CMVs group, whereas it did not decrease in the ASV group. They concluded that ASV may protect the diaphragm against the deleterious effect of prolonged PC-CMVs | [10] |

Table 5: Animal studies of MFV

| Author | Year | Population | Study Purpose | Conclusions |  |
| --- | --- | --- | --- | --- | --- |
| Mireles-Cabodevila | 2014 | 6 pigglets | Compared the effects of PC-CMVs using a lung-protective strategy with MFV in a porcine model of lung injury. | Demonstrated that MFV allows the use of higher breathing frequencies and lower tidal volumes than conventional ventilation to maximize alveolar ventilation. There were no significant differences in any hemodynamic measurements, although heart rate was higher during MFV. Also showed that the application of a decision support algorithm to manage MFV is feasible. | [83] |

Table 6: Human studies of MFV

| Author | Year | Population | Study Purpose | Conclusions |  |
| --- | --- | --- | --- | --- | --- |
| Bhat | 2017 | 12 preterm infants with respiratory distress syndrome (RDS) | Randomized controlled study comparing MFV to PC-IMVs | MFV allowed the use of lower inspiratory pressures and tidal volumes while maintaining adequate gas exchange. Peak inspiratory pressure and tidal volume, the surrogate indicators of volutrauma and barotrauma, were lower during mid-frequency ventilation. | [11] |

## AVM 2

AVM 2 was announced in 2017[12]. van der Staay and Remus compared AVM 2 with AVM and ASV and demonstrated with a test lung that minimizing inspiratory power (AVM 2) results in higher frequencies, lower inspiratory pressures, and lower V_T_ compared with minimizing breathing power (AVM and ASV). The V_T_/kg ratio dropped from 7 mL/kg to 5.3 mL/kg for a test lung with lung restrictive characteristics. However, there are currently no animal or human studies available with AVM 2.

# Theoretical Comparison of ASV, MFV, and AVM 2

When comparing these modes with others, we will refer to them as ASV, AVM2, and MFV for simplicity and to be consistent with the above descriptions. But it is helpful to recognize the classification of the modes[2] when comparing them to more conventional modes (which have a much wider variety of names). Hence, ASV and AVM 2 are both classified as PC-IMVoi,oi. This means they are forms of pressure control intermittent mandatory ventilation that use both optimal (o) and intelligent (i) targeting schemes for mandatory and spontaneous breaths (intelligent targeting schemes use tools of artificial intelligence, such as rule-based expert systems, fuzzy logic, and artificial neural networks). For these two modes, optimal targeting automatically selects tidal volume and frequency targets based on lung mechanics, while expert rules enforce safety limitations on those targets. MFV, in the form reported in the literature, is PC-IMVo,s which indicates that mandatory breaths use optimal targeting while spontaneous breaths use set-point (s) targeting (ie, no targets are automatically set by the ventilator). The patent for MFV describes the use of artificial intelligence but the mode has not yet been commercialized.

The optimal targeting schemes described above have one thing in common: for a given required alveolar minute ventilation and set of lung mechanics (for passive ventilation), they all suggest a ventilatory frequency that is optimal in some way (along with the associated optimal tidal volume). For a given alveolar minute ventilation, there are only two parameters which are free to vary, (alveolar volume and frequency), and there are virtually infinite combinations. These targeting schemes offer three different ways to make the optimal selections. ASV is based on a model of *unassisted* breathing (sinusoidal pressure waveform driving function), under the assumption that the optimal tidal volume and frequency are those that would be picked by the patient’s brain to minimize power output of the muscles. AVM 2 is based on a model of *assisted* breathing (square pressure waveform driving function) and selects tidal volume and frequency such that inspiratory power will be minimized to possibly avoid ventilator induced lung injury. Likewise, MFV was invented with the intention of serving lung protective ventilation by choosing the frequency that minimizes inspiratory pressure. Table 7 compares these modes.

Currently, there is intensive research in the field of ventilation induced lung injury, but answers are still pending[84]. A further complication is the fact that the goals of mechanical ventilation (safety, comfort, and liberation) are often mutually exclusive. Hence, we did not seek to rate the performance of the optimum targeting schemes in terms of clinical outcomes because this is not possible without further experimental evidence. Instead, we determined how these targeting schemes behaved during selected simulation scenarios. This may promote understanding on a more intuitive level, instead of analyzing abstract mathematical equations.

Table 7: Conceptual comparison of ASV, AVM 2 and Mid-Frequency Ventilation. They use different models and targeting schemes to choose optimal settings of tidal volume and respiratory rate.

|  | ASV | AVM 2 | Mid-Frequency |
| --- | --- | --- | --- |
| Optimization Target: | Minimal Breathing Power | Minimal Inspiratory Power | Minimal inspiratory pressure or maximal alveolar minute ventilation |
| Ventilation Model: | Spontaneously breathing without ventilator. | Pressure controlled ventilation with square waveform | Pressure controlled ventilation with square waveform |
| Lung Model: | One compartment, linear R and C | One compartment, linear R and C | One compartment, linear R and C, different values for R_I_ and R_E_ |
| Calculation: | Numerical, recursive | Numerical, recursive | Experimental |

## Simulation Parameters

We performed a comparison of the three targeting schemes by assuming four different mathematical simulation scenarios. The values of resistance and compliance were based on the work of Arnal et al[85] for adult patients, and of Ellen et al [86] for the neonatal scenario. With regard to dead space (V_D_), there are several options. The physiological dead space, based on the Bohr equation (V_D-B_), is consistent with the equation V_T_ = V_A_ + V_D_.[87] ASV and AVM 2 targeting schemes assume the dead space to be anatomical (V_D-A_), which is estimated to be 2.2 mL/kg[88] among all patient types regardless of the condition. In the ICU, V_D_/V_T_ calculated with the blood gas measurement of PaCO_2_ is a useful indicator for the efficiency of ventilation. In this case, the dead space is calculated with the Enghoff modification of the Bohr equation (V_D-E_). Note, that this volume does not necessarily exist physically and usually overestimates the physiological dead space volume V_D-B_ (thus it underestimates the required ${\dot{\text{V}}}_{\text{A}}$)[89]. To be clear, the presence of shunt and low $\dot{\text{V}}/\dot{\text{Q}}$ are not dead space volumes but their effects manifest in the form of “virtual” dead space ( the difference betweenV_D-E_ andV_D-B_). Hence, if we want to simulate clinical experience using actual modes, we use V_D-A_ for the frequency calculation of ASV and AVM 2, respectively, and V_D-E_ for MFV. However, we want to compare these targeting schemes at the same level of simulated PaCO_2_. For this reason we define the “Enghoff alveolar minute volume” as

|  | ${\dot{\text{V}}}_{\text{A-E}}=f\cdot\left( V_{T}-V_{\text{D-E}} \right)$ | (35) |
| --- | --- | --- |

which may underestimate the alveolar minute volume ${\dot{\text{V}}}_{\text{A}}$ but is best correlated to PaCO_2_. This reflects practical ventilation performance and efficiency as realistic as possible.

We assumed for adult ARDS simulation V_D-E_ = 4.4 mL/kg[90], for normal adults V_D-E_ = 2.2 mL/kg and for adult COPD V_D-E_/V_T_ = 0.49[91] at V_T_/kg = 8.9 mL/kg[85] which results also in V_D-E_ = 4.4 mL/kg. For Neonatal scenario we used for normal infants V_D-E_ = 2.5 mL/kg[86], for RDS scenario V_D-E_ = 3.8 mL/kg[86] and for CLD scenario V_D-E_ = 3.8 mL/kg[86].

The parameters of the lung models are listed in Table 8. Furthermore, we assumed passive inspiration and a linear one compartment lung model with airway resistance R = R_I_ = R_E_. The ventilator inspiration to expiration ratio was assumed to be 1:1. With these assumptions, we calculated the optimum frequency with the equation (20) for ASV, equation (27) for AVM 2 and equation (33) for MFV and then derived tidal volume (V_T_ = ${\dot{\text{V}}}_{\text{A}}$/f+V_D_), tidal pressure (V_T_/C), tidal power (f·V_T_·P_T_/2) and Enghoff alveolar minute volume ${\dot{\text{V}}}_{\text{A-E}}$ by equation (35).

Table 8: Patient characteristics for different scenarios. The anatomic dead space V_D-A_ was used for the frequency calculation of ASV and AVM 2. V_D-E_ represents the dead space determined by PaCO_2_ and the Enghoff modification of the Bohr equation. For the simulation, this was used both for the frequency calculation of MFV and for the calculation of the Enghoff alveolar minut volume ${\dot{\text{V}}}_{\text{A-E}}$.

| Scenario | Ideal Weight | R | C | V_D-A_ | V_D-E_ |
| --- | --- | --- | --- | --- | --- |
| Adult: | 70 kg | 10 cmH_2_O /l/s | 55 ml/cmH_2_O | 154 ml | 154 ml |
| Adult COPD: | 70 kg | 20 cmH_2_O/l/s | 60 ml/cmH_2_O | 154 ml | 308 ml |
| Adult Severe ARDS: | 70 kg | 10 cmH_2_O /l/s | 35 ml/cmH_2_O | 154 ml | 308 ml |
| Neonatal: | 1 kg | 37 cmH_2_O /l/s | 1.5 ml/cmH_2_O | 2.2 ml | 2.5 ml |
| Neonatal RDS: | 1 kg | 120 cmH_2_O /l/s | 0.4 ml/cmH_2_O | 2.2 ml | 3.8 ml |
| Neonatal CLD: | 1 kg | 90 cmH_2_O /l/s | 0.5 ml/cmH_2_O | 2.2 ml | 3.8 ml |

Calculation of the optimum frequency for the MFV target scheme was done experimentally using different simulated ventilator frequency setting values. We chose an interval of 0.1 breaths per minute to avoid excessive error. The same precision was applied for the iterative calculation of the ASV and AVM 2 frequency. The simulation was done with the software package Matlab from Mathworks and comprises following steps:

- 1. Set target alveolar minute volume MV_A_
  2. Calculate and find the optimal frequency for ASV, AVM2 and MFV by the equations of the first section and the parameters defined above.
  3. Calculate the resultant ventilation parameters of tidal volume, tidal pressure, tidal power, inspiratory power and Enghoff alveolar minute volume for comparison.
  4. Repeat steps a) to c) for different alveolar minute volume

## Adults

For adult 70 kg simulation, we compare normal, ARDS and COPD lung characteristics according to Table 1. Results are shown in Figure 12. The green area highlights the range which is used normaly for this patients according to Arnal et al[32].

## Neonatal

For neonatal simulation, we compare RDS and normal lung characteristics according to Table 8. Results are shown in Figure 11. The green area highlights the range which is used normaly for this patients according to McCann et al [33].


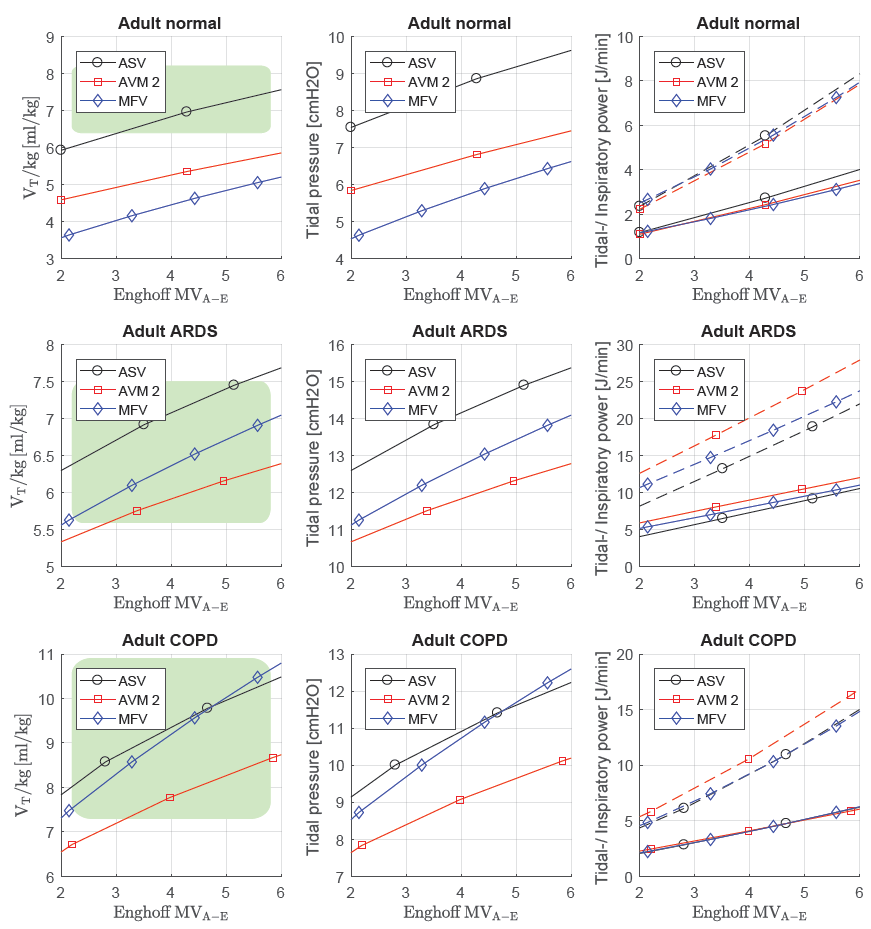


Figure 12: Simulation results of adult normal, severe ARDS and COPD. Data points are optimum values for tidal volume, tidal pressure, tidal power (solid lines) and inspiratory power (dashed lines) for various levels of Enghoff alveolar minute ventilation (MV_A-E_). The green area highlights the area which is normaly used according to the work of Arnal. For COPD, ASV and MFV have similar results. For ARDS simulation scenario, the concept of AVM 2 yields the lowest tidal volumes and the concept of ASV uses highest tidal volumes.


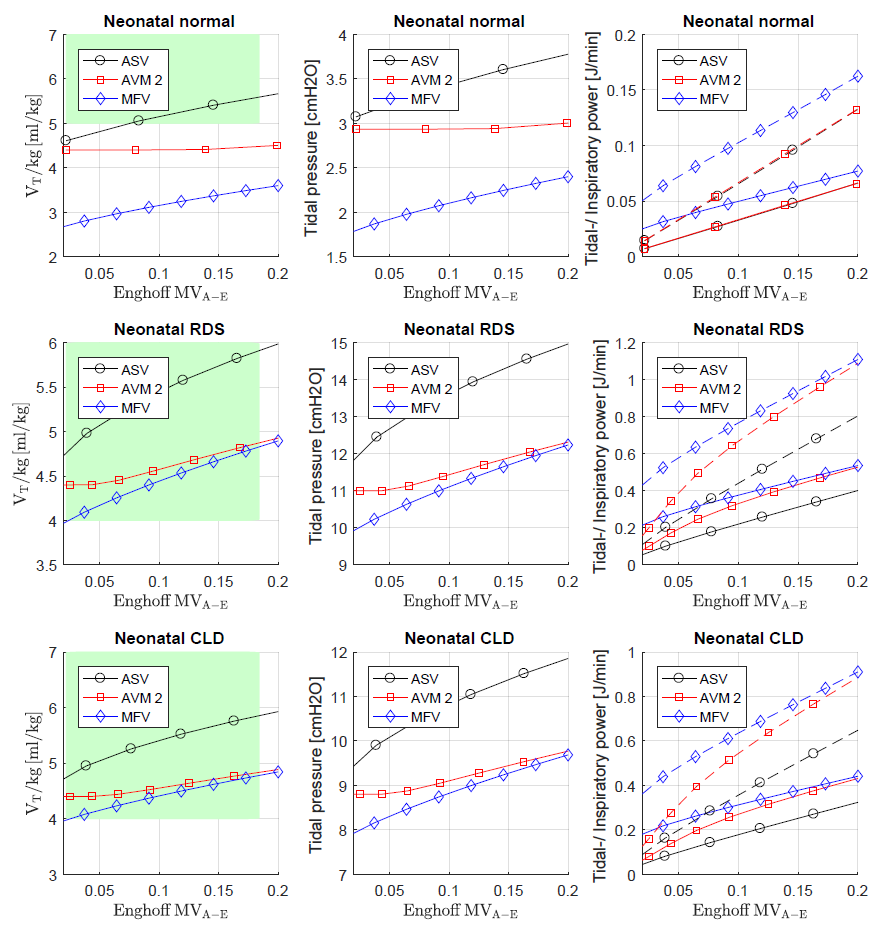


Figure 13: Simulation results of neonatal normal, RDS and CLD. Data points are optimum values for tidal volume, tidal pressure, tidal power (solid lines) and inspiratory power (dashed lines) for various levels of Enghoff alveolar minute ventilation (MV_A-E_). The green area highlights the area which is normaly used according to the work of McCann. For AVM 2, it can be seen, that the V_T_/kg ratio does not fall below 2·V_D-A_, which is the equivalent for minimal tidal power. For RDS, CLD and normal simulation scenario, the concept of MFV yields the lowest tidal volumes and the concept of ASV yields the highest tidal volumes.

## Discussion

### The Effect of Dead Space

An attentive observer of Figure 12 and Figure 13 will notice that AVM 2, which optimizes by minimizing inspiratory power, does not always have the lowest inspiratory power. On the other hand MFV, which optimizes by minimizing inspiratory pressure, does not always have the smallest tidal pressure. These findings can be explained by the mismatch of dead space V_D_. For ASV and AVM2, the calculation of the optimal frequencies are based on V_D-A_ and not on V_D-E_. Therefore, if V_D-A_ is significantly lower than V_D-E_, the Enghoff alveolar minute volume is underestimated. To get the same Enghoff alveolar minute volume, we increased the alveolar minute volume until the desired Enghoff alveolar minute volume was reached (resulting in the same simulated PaCO_2_ level). Hence, the calculation for the optimal frequencies for ASV, AVM2 and MFV cannot be done at the same alveolar minute volume to reach the same Enghoff alveolar minute volume. If V_D-E_ is used for the optimal frequency calculation of ASV and AVM2, this mismatch will not occur, and AVM2 will have the lowest inspiratory power compared to ASV and MFV.

Given the assumption that V_D_ = 2.2 mL/kg, the initial settings using these targeting schemes will likely result in hypoventilation when actually ventilating patients with increased V_D_ (like ARDS and COPD. If, on the other hand, a factor based on measured physiologic V_D_ is used, then there's a greater likelihood that the initial result will be within the target range for CO_2_. The main point of interest for clinicians relates to modes that require manual adjustment of the minute ventilation target (eg, all conventional modes, ASV and AVM1). For these modes, under-estimating the required minute alveolar ventilation by use of an estimate of V_D_ that is too small when making initial ventilator settings (frequency and tidal volume) will result in subsequent manual adjustments and delay in achieving the PaCO_2_ target. For modes that automatically adjust the minute ventilation using ongoing monitoring of etPCO_2_, subsequent adjustments are probably not as important an issue, but may still delay achievement of the desired PaCO_2_.

Nevertheless, the simulation reflects the practical experience as realistically as possible because we have the same mismatch in reality. We believe that optimal targeting schemes might be more “optimal”, in terms of their optimization target, if they used V_D-B_ instead of V_D-A_ or V_D-E_. Otherwise they have a mismatch between the model (V_T_=V_A_+V_D_) and reality. On the other hand, there is clinical evidence that these targeting schemes work, with a few exceptions, for most lung conditions. If they are applied and understood correctly in practice, they may simplify clinical practice.

### Optimal Ventilation and VILI

In the first section of this work, we analyzed analytically, how ventilation would look like if the clinical indicators Tidal Volume, Tidal Pressure, Tidal Power or Driving Power were stringently optimized. For Tidal Volume and Tidal Pressure, the solution would be in the range of V_T_ ~ V_D_ at the highest possible frequency. At least for the ventilation of adults, this value seems to be far beyond the usual (green area in the figures). Also for the condition of minimal tidal power and driving power (V_T_ = 2·V_D_), V_T_ seems too low for normal lungs. But this does not necessarily mean that they are bad predictors for VILI. On the other hand, we observe from Figure 12 and Figure 13 that the inspiratory power for the adult simulations is more than a decade higher than that of the neonatal scenarios. As Marini already observed, adjustment to the reduced baby lung capacity may be necessary. This indicates that the cost function, which discribes optimal ventilation, does not have to be necessarily a good predictor for VILI. Therefore, the answer to the questions “*What should we optimize?*” and “*What induces lung injuries?*” might be not the same. However, it seems reasonable to understand that a cost function describing optimal ventilation should approach, at least for patients at risk for lung injuries, the cost function that describes VILI.

We see from the simulation, that the results are supporting Marini’s proposal to adjust an indicator for VILI for compliance (as mentioned above). This concept also seems to be confirmed (at least in a mathematical sense) by the fact that tidal pressure (driving pressure) can be interpreted as a compliance adjusted tidal volume. Finally, the comparison between neonatal and adult scenarios suggests that tidal pressure is more related to the lung conditions than to the weight of the patient.

### Limitations

For the simulations, we applied some simplifying assumptions and we did not include specific details reflectiong the actual ventilator mode implementations of the targeting schemes. For example, ASV and AVM2 each have multiple “expert rules”, which could limit the frequency or tidal volume in certain scenarios (eg, to avoid large tidal volumes or high intrinsic PEEP). Also, ASV is currently not designed for neonatal ventilation and limits its frequency to between 5 and 60 breaths per minute. Furthermore, AVM2 actually assumes an I:E ratio of 1:1.8 , not 1:1, which was empirically derived during design of the mode implementation. This would lead to higher tidal volumes for normal lung conditions. MFV is designed to allow accommodation of unequal inspiratory and expiratory airway resistances.

# Conclusion

Modes of mechanical ventilation have shown a steady evolution over the last 4 decades. They have increased in complexity as engineers attempt to add technical capabilities that better serve clinical goals. A key feature of this complexity is the development of new targeting schemes, moving away from simple set-point targeting (all targets are operator preset) through adaptive targeting (some targets are automatically adjusted) to optimal targeting (targets are automatically adjusted to maximize or minimize some desired performance characteristic) and even intelligent targeting (automatic adjustment and selection of targets using the tools of artificial intelligence). In particular, optimum targeting schemes have been the central feedback control mechanisms of the most complex modes currently available. Optimization means, by definition, that there exists no better alternative to get, do or set something, given the constraints of the mathematical model used. But we have shown that this kind of targeting scheme for ventilator modes is based on fairly arbitrary assumptions and presupposes clearly defined goals and targets, which are still topics of clinical debate. This paper tries to clarify these assmptions and point out that thinking about *what* should be optimized is much more important than thinking about *how* we should optimize. We suggest that optimization based on tidal volume, tidal pressure, or tidal power as the sole criteria, may result in unusual ventilation strategies and settings. Therefore, we can adumbrate that modes based only on one of these variables may have limited clinical success.

# References

1. Moore GE (1998) Cramming more components onto integrated circuits. Proceedings of the IEEE 86(1):82–85

2. Chatburn RL, El-Khatib M, Mireles-Cabodevila E (2014) A taxonomy for mechanical ventilation: 10 fundamental maxims. Respiratory care 59(11):1747–1763

3. Chatburn RL (2004) Computer control of mechanical ventilation. Respiratory care 49(5):507–517

4. Chatburn RL, Mireles-Cabodevila E (2011) Closed-loop control of mechanical ventilation: description and classification of targeting schemes. Respiratory care 56(1):85–102

5. Wysocki M, Brunner JJX (2007) Closed-Loop Ventilation: An Emerging Standard of Care? Critical Care Clinics 23(2):223–40, ix

6. Tehrani FT (2008) Automatic control of mechanical ventilation. Part 1: Theory and history of the technology. Journal of Clinical Monitoring and Computing 22(6):409–415

7. Otis B (1954) The work of Breathing. Physiol Rev 34(3):449–458

8. Gattinoni L, Tonetti T, Cressoni M, et al (2016) Ventilator-related causes of lung injury: the mechanical power. Intensive Care Medicine 42(10):1567–1575

9. Marini JJ, Jaber S (2016) Dynamic predictors of VILI risk: beyond the driving pressure. Intensive Care Medicine 42(10):1597–1600

10. Mireles-Cabodevila E, Chatburn RL (2008) Original Contributions Mid-Frequency Ventilation : Unconventional Use of Conventional Mechanical Ventilation as a Lung-Protection Strategy. Respiratory care 53(12):1669–1677

11. Bhat R, Kelleher J, Ambalavanan N, et al (2017) Feasibility of Mid-Frequency Ventilation Among Infants With Respiratory Distress Syndrome. Respiratory Care 62(4):481–488

12. van der Staay M, Remus C (2017) Adaptive Ventilation Mode 2. https://downloads.imt.ch/usdavkarsv/scientificNote_AVM2.pdf. Accessed 12 Mar 2018

13. Crooke PS, Head JD, Marini JJ (1996) A general two-compartment model for mechanical ventilation. Mathematical and Computer Modelling 24(7):1–18

14. Crooke PS, Head JD, Marini JJ, Hotchkiss JR (1998) Patient-ventilator interaction: a general model for nonpassive mechanical ventilation. IMA journal of mathematics applied in medicine and biology 15(4):321–37

15. Marini JJ, Crooke PS, Truwit JD (1989) Determinants and limits of pressure-preset ventilation: a mathematical model of pressure control. Journal of applied physiology (Bethesda, Md : 1985) 67(3):1081–92

16. Marini JJ, Crooke PS, 3rd (1993) A General Mathematical model for Respiratory Dynamics Relevant to the Clinical Setting. The American review of respiratory disease 147(1):14–24

17. Brower RE Al (2000) Ventilation With Lower Tidal Volumes As Compared With Traditional Tidal Volumes for Acute Lung Injury and the Acute Respiratory Distress Syndrome. The New England journal of medicine 342(18):1301–1308

18. Bowton DL, Scott LK (2016) Ventilatory Management of the Noninjured Lung. Clinics in Chest Medicine 37(4):701–710

19. Serpa Neto A, Nagtzaam L, Schultz MJ (2014) Ventilation with lower tidal volumes for critically ill patients without the acute respiratory distress syndrome: a systematic translational review and meta-analysis. Current opinion in critical care 20(1):25–32

20. Neto AS, Simonis FD, Barbas CS, et al (2015) Lung-Protective Ventilation With Low Tidal Volumes and the Occurrence of Pulmonary Complications in Patients Without Acute Respiratory Distress Syndrome: A Systematic Review and Individual Patient Data Analysis. Crit Care Med 43(10):2155–2163

21. Serpa Neto A, Simonis FD, Schultz MJ (2015) How to ventilate patients without acute respiratory distress syndrome? Current Opinion in Critical Care 21(1):65–73

22. Brochard L, Slutsky A, Pesenti A (2017) Mechanical ventilation to minimize progression of lung injury in acute respiratory failure. American Journal of Respiratory and Critical Care Medicine 195(4):438–442

23. Chatburn RL, Mireles-Cabodevila E (2011) Handbook of Respiratory Care

24. Driving Pressure and Survival in the Acute Respiratory Distress Syndrome. http://intensivo.sochipe.cl/subidos/catalogo3/Driving Presuure in SDRA NEJM 2015.pdf. Accessed 29 Apr 2015

25. Chiumello D, Carlesso E, Brioni M, Cressoni M (2016) Airway driving pressure and lung stress in ARDS patients. Critical Care 20(1):276

26. Borges JB, Hedenstierna G, Larsson A, Suarez-Sipmann F (2015) Altering the mechanical scenario to decrease the driving pressure. Critical Care 19(1):342

27. Tejerina E, Pelosi P, Muriel A, et al (2017) Association between ventilatory settings and development of acute respiratory distress syndrome in mechanically ventilated patients due to brain injury. Journal of Critical Care 38:341–345

28. Serpa Neto A, Schmidt M, Azevedo LCP, et al (2016) Associations between ventilator settings during extracorporeal membrane oxygenation for refractory hypoxemia and outcome in patients with acute respiratory distress syndrome: a pooled individual patient data analysis: Mechanical ventilation during ECMO. Intensive Care Medicine 42(11):1672–1684

29. Neto AS, Hemmes SNT, Barbas CS V, et al (2016) Association between driving pressure and development of postoperative pulmonary complications in patients undergoing mechanical ventilation for general anaesthesia: A meta-analysis of individual patient data. The Lancet Respiratory Medicine 4(4):272–280

30. Guérin C, Papazian L, Reignier J, et al (2016) Effect of driving pressure on mortality in ARDS patients during lung protective mechanical ventilationin two randomized controlled trials. Critical Care 20(1):384

31. Cressoni M, Gotti M, Chiurazzi C, Massari D, Algieri I, Amini M, Cammaroto A, Brioni M, Montaruli C, Nikolla K, Guanziroli M, Dondossola D, Gatti S, Valerio V, Vergani GL, Pugni P, Cadringher P, Gagliano N GL, Gattinoni L, Gagliano N, et al (2016) Mechanical Power and Development of Ventilator-induced Lung Injury. Anesthesiology 124(5):1100–1108

32. Otis AB, Fenn WO, Rahn H (1950) Mechanics of breathing in man. Journal of applied physiology 2(11):592–607

33. Mead J (1960) Control of respiratory frequency. Journal of Applied Physiology 15(3):325–336

34. Laubscher TP, Frutiger A, Fanconi S, Brunner JX (1996) The automatic selection of ventilation parameters during the initial phase of mechanical ventilation. Intensive Care Medicine 22(3):199–207

35. Laubscher TP, Frutiger A, Fanconi S, et al (1994) Automatic selection of tidal volume, respiratory frequency and minute ventilation in intubated ICU patients as startup procedure for closed-loop controlled ventilation. International Journal of Clinical Monitoring and Computing 11(1):19–30

36. Dongelmans DA, Veelo DP, Bindels A, et al (2008) Determinants of Tidal Volumes with Adaptive Support Ventilation: A Multicenter Observational Study. Anesthesia & Analgesia 107(3):932–937

37. Villar J, Kacmarek RM, Hedenstierna G (2004) From ventilator-induced lung injury to physician-induced lung injury: Why the reluctance to use small tidal volumes? Acta Anaesthesiol. Scand. 48:267–271

38. Sulemanji D, Kacmarek R (2010) Adaptive support ventilation: an inappropriate mechanical ventilation strategy for acute respiratory distress syndrome? Anesthesiology 111(5):1295–1296

39. Arnal J-M, Garnero A, Novonti D, et al (2013) Feasibility study on full closed-loop control ventilation (IntelliVent-ASV^TM^) in ICU patients with acute respiratory failure: a prospective observational comparative study. Critical Care 17(5):R196

40. Amato MBP, Meade MO, Slutsky AS, et al (2015) Driving Pressure and Survival in the Acute Respiratory Distress Syndrome. New England Journal of Medicine 372(8):747–755

41. Hewlett a M, Platt a S, Terry VG (1977) Mandatory minute volume. A new concept in weaning from mechanical ventilation. Anaesthesia 32(2):163–169

42. Laubscher T P, W H, N W, et al (1994) An Adaptive lung Ventilation Contoller. IEEE Transactions on Biomedical Engineeringmedical Engineering 41(1):51–58

43. Belliato M, Palo A, Pasero D, et al (2004) Evaluation of adaptive support ventilation in paralysed patients and in a physical lung model. International Journal of Artificial Organs 27(8):709–716

44. Sulemanji D, Marchese A, Garbarini P, et al (2009) Adaptive support ventilation: an appropriate mechanical ventilation strategy for acute respiratory distress syndrome? Anesthesiology 111(4):863–870

45. Veelo DP, Dongelmans DA, Binnekade JM, et al (2010) Adaptive support ventilation: A translational study evaluating the size of delivered tidal volumes. International Journal of Artificial Organs 33(5):302–309

46. Morato JB, Sakuma MTA, Ferreira JC, Caruso P (2012) Comparison of 3 modes of automated weaning from mechanical ventilation: A bench study. Journal of Critical Care 27(6):741.e1-741.e8

47. Mireles-Cabodevila E, Diaz-Guzman E, Arroliga AC, Chatburn RL (2012) Human versus computer controlled selection of ventilator settings: An evaluation of adaptive support ventilation and mid-frequency ventilation. Critical Care Research and Practice 2012

48. Sulemanji DS, Marchese A, Wysocki M, Kacmarek RM (2013) Adaptive support ventilation with and without end-tidal CO2 closed loop control versus conventional ventilation. Intensive Care Medicine 39(4):703–710

49. Jung B, Constantin J-M, Rossel N, et al (2010) Adaptive support ventilation prevents ventilator-induced diaphragmatic dysfunction in piglet: an in vivo and in vitro study. Anesthesiology 112(6):1435–1443

50. Laubscher TP, Heinrichs W, Weiler N, et al (1994) An adaptive lung ventilation controller. IEEE Transactions on Biomedical Engineering 41(1):51–59

51. Weiler N, Heinrichs W, Keßler W (1994) The AVL-mode: A safe closed loop algorithm for ventilation during total intravenous anesthesia. International Journal of Clinical Monitoring and Computing 11(2):85–88

52. Linton DM, Potgieter PD, Davis S, et al (1994) Automatic weaning from mechanical ventilation using an adaptive lung ventilation controller. Chest 106(6):1843–1850

53. Weiler N, Eberle B, Heinrichs W (1998) Adaptive lung ventilation (ALV) during anesthesia for pulmonary surgery: Automatlc response to transitions to and from one-lung ventilation. Journal of Clinical Monitoring and Computing 14(4):245–252

54. Sulzer CF, Chioléro R, Chassot PG, et al (2001) Adaptive support ventilation for fast tracheal extubation after cardiac surgery: a randomized controlled study. Anesthesiology 95(6):1339–45

55. Tassaux D, Dalmas E, Gratadour P, Jolliet P (2002) Patient-ventilator interactions during partial ventilatory support: a preliminary study comparing the effects of adaptive support ventilation with synchronized intermittent mandatory ventilation plus inspiratory pressure support. Critical care medicine 30(4):801–7

56. Cassina T, Chioléro R, Mauri R, Revelly J-P (2003) Clinical experience with adaptive support ventilation for fast-track cardiac surgery. Journal of cardiothoracic and vascular anesthesia 17(5):571–5

57. Petter AH, Chioléro RL, Cassina T, et al (2003) Automatic “Respirator/Weaning” with Adaptive Support Ventilation: The Effect on Duration of Endotracheal Intubation and Patient Management. Anesthesia & Analgesia 97(6):1743–1750

58. Linton DM, Renov G, Lafair J, et al (2006) Adaptive Support Ventilation as the sole mode of ventilatory support in chronically ventilated patients. Critical care and resuscitation : journal of the Australasian Academy of Critical Care Medicine 8(1):11–14

59. Arnal J-M, Wysocki M, Nafati C, et al (2008) Automatic selection of breathing pattern using adaptive support ventilation. Intensive care medicine 34(1):75–81

60. Gruber PC, Gomersall CD, Leung P, et al (2008) Randomized controlled trial comparing adaptive-support ventilation with pressure-regulated volume-controlled ventilation with automode in weaning patients after cardiac surgery. Anesthesiology 109(1):81–87

61. Dongelmans DA, Veelo DP, Bindels A, et al (2008) Determinants of tidal volumes with adaptive support ventilation: A multicenter observational study. Anesthesia and Analgesia 107(3):932–937

62. Veelo DP, Dongelmans DA, Middelhoek P, et al (2008) Adaptive support ventilation with percutaneous dilatational tracheotomy: A clinical study. Anesthesia and Analgesia 107(3):938–940

63. Dongelmans DA, Veelo DP, Paulus F, et al (2009) Weaning automation with adaptive support ventilation: A randomized controlled trial in cardiothoracic surgery patients. Anesthesia and Analgesia 108(2):565–571

64. Lloréns J, Ballester M, Tusman G, et al (2009) Adaptive support ventilation for gynaecological laparoscopic surgery in Trendelenburg position: bringing ICU modes of mechanical ventilation to the operating room. European Journal of Anaesthesiology 26(2):135–139

65. Jaber S, Sebbane M, Verzilli D, et al (2009) Adaptive support and pressure support ventilation behavior in response to increased ventilatory demand. Anesthesiology 110(3):620–7

66. Dongelmans DA, Veelo DP, Binnekade JM, et al (2010) Adaptive support ventilation with protocolized de-escalation and escalation does not accelerate tracheal extubation of patients after nonfast-track cardiothoracic surgery. Anesthesia and Analgesia 111(4):961–967

67. Iotti GA, Polito A, Belliato M, et al (2010) Adaptive support ventilation versus conventional ventilation for total ventilatory support in acute respiratory failure. Intensive Care Medicine 36(8):1371–1379

68. Dongelmans DA, Paulus F, Veelo DP, et al (2011) Adaptive support ventilation may deliver unwanted respiratory rate-tidal volume combinations in patients with acute lung injury ventilated according to an open lung concept. Anesthesiology 114(5):1138–1143

69. Chen C-W, Wu C-P, Dai Y-L, et al (2011) Effects of implementing adaptive support ventilation in a medical intensive care unit. Respiratory care 56(7):976–983

70. Kirakli C, Ozdemir I, Ucar ZZ, et al (2011) Adaptive support ventilation for faster weaning in COPD: a randomised controlled trial. European Respiratory Journal 38(4):774–780

71. Arnal J-M, Wysocki M, Novotni D, et al (2012) Safety and efficacy of a fully closed-loop control ventilation (IntelliVent-ASV®) in sedated ICU patients with acute respiratory failure: a prospective randomized crossover study. Intensive care medicine 38(5):781–7

72. Agarwal R, Srinivasan A, Aggarwal AN, Gupta D (2013) Adaptive support ventilation for complete ventilatory support in acute respiratory distress syndrome: A pilot, randomized controlled trial. Respirology 18(7):1108–1115

73. Celli P, Privato E, Ianni S, et al (2014) Adaptive support ventilation versus synchronized intermittent mandatory ventilation with pressure support in weaning patients after orthotopic liver transplantation. Transplantation Proceedings 46(7):2272–2278

74. Han L, Wang Y, Gan Y, Xu L (2014) Effects of Adaptive Support Ventilation and Synchronized Intermittent Mandatory Ventilation on Peripheral Circulation and Blood Gas Markers of COPD Patients with Respiratory Failure. Cell Biochem. Biophys. 70:481–484

75. Ng S, Gomersall C, Zhu F, et al (2015) A Randomized Controlled Trial of Adaptive Support Ventilation Mode to Wean Patients after Fast-track Cardiac Valvular Surgery. Anesthesiology 122(4):1–9

76. Kirakli C, Naz I, Ediboglu O, et al (2015) A randomized controlled trial comparing the ventilation duration between Adaptive Support Ventilation and Pressure Assist/Control Ventilation in medical ICU patients. Chest 147(6):1503–1509

77. Yazdannik A, Zarei H, Massoumi G (2016) Comparing the effects of adaptive support ventilation and synchronized intermittent mandatory ventilation on intubation duration and hospital stay after coronary artery bypass graft surgery. Iranian Journal of Nursing and Midwifery Research 21(2):207

78. Tam MKP, Wong WT, Gomersall CD, et al (2016) A randomized controlled trial of 2 protocols for weaning cardiac surgical patients receiving adaptive support ventilation. Journal of Critical Care 33:163–168

79. Ghodrati M, Pournajafian A, Khatibi A, et al (2016) Comparing the effect of adaptive support ventilation (ASV) and synchronized intermittent mandatory ventilation (SIMV) on respiratory parameters in neurosurgical ICU patients. Anesthesiology and Pain Medicine 6(6)

80. Moradian ST, Saeid Y, Ebadi A, et al (2017) Adaptive support ventilation reduces the incidence of atelectasis in patients undergoing coronary artery bypass grafting: A randomized clinical trial. Anesthesiology and Pain Medicine 7(3)

81. Kiaei BA, Kashefi P, Hashemi ST, et al (2017) The Comparison Effects of Two Methods of (Adaptive Support Ventilation Minute Ventilation: 110% and Adaptive Support Ventilation Minute Ventilation: 120%) on Mechanical Ventilation and Hemodynamic Changes and Length of Being in Recovery in Intensive Care . Advanced biomedical research 6:52

82. Peng CK, Wu SF, Yang SH, et al (2017) Correlation between transition percentage of minute volume (TMV%) and outcome of patients with acute respiratory failure. Journal of Critical Care 39:178–181

83. Mireles-Cabodevila E, Chatburn RL, Thurman TL, et al (2014) Application of mid-frequency ventilation in an animal model of lung injury: a pilot study. Respiratory care respcare-03105

84. Marini JJ (2018) Dissipation of energy during the respiratory Cycle: Conditional importance of ergotrauma to structural lung damage. Current Opinion in Critical Care 24(1):16–22

85. Arnal J-M, Garnero A, Saoli M, Chatburn RL (2018) Parameters for Simulation of Adult Patients During Mechanical Ventilation. Respiratory Care 63(2):158 LP-168

86. McCann EM, Goldman SL, Brady JP (1987) Pulmonary function in the sick newborn infant. Pediatric Research 21(4):313–325

87. Suarez-Sipmann F, Bohm SH, Tusman G (2014) Volumetric capnography: The time has come. Current Opinion in Critical Care 20(3):333–339

88. Radford EP, Ferris BG, Kriete BC (1954) Clinical use of a nomogram to estimate proper ventilation during artificial respiration. The New England journal of medicine 251(22):877–884

89. Tusman G, Sipmann FS, Bohm SH (2012) Rationale of dead space measurement by volumetric capnography. Anesthesia and Analgesia 114(4):866–874

90. Kallet RH, Zhuo H, Ho K, et al (2017) Lung Injury Etiology and Other Factors Influencing the Relationship Between Dead-Space Fraction and Mortality in ARDS. Respiratory care 62(10):1241–1248

91. Farah R, Makhoul N (2009) Can dead space fraction predict the length of mechanical ventilation in exacerbated COPD patients? International journal of chronic obstructive pulmonary disease 4:437–441
